# Towards modelling mental disorders in zebrafish. Neurexins severely modulate anxiety, affiliative social behaviour and aggression

**DOI:** 10.1038/s41380-026-03721-1

**Published:** 2026-06-26

**Authors:** Q. Nguyen, F. Guo, J. Das, O. Hatzimanolis, B. Mowry, Alexandre S. Cristino, J. Giacomotto

**Affiliations:** 1 Institute for Biomedicine and Glycomics, Griffith University, Brisbane, QLD 4111 Australia; 2 Queensland Brain Institute, The University of Queensland, Brisbane, QLD 4072 Australia; 3 School of Environment and Science, Griffith University, Brisbane, QLD 4111 Australia; 4 Thomson Institute, National PTSD Research Centre, University of the Sunshine Coast, Birtinya, QLD Australia

**Keywords:** Schizophrenia, Autism spectrum disorders, Depression, Genetics, Neuroscience

## Abstract

Neurexin (*nrxn*) genes encode synaptic cell-adhesion molecules that have been repetitively associated with neurodevelopmental and mental disorders. While the zebrafish animal model offers tremendous advantages for dissecting neural development/function, no complete loss-of-function (LOF) *nrxn* zebrafish models are currently available. In this study, we generated the first collection of zebrafish knockout lines for each *nrxn* gene, with mutations ranging from transmembrane domain- to full genomic locus-deletions. Surprisingly, all homozygous lines developed normally, presenting no gross neurodevelopmental or obvious early behavioural abnormalities. However, this absence of early phenotypes translated into profound, paralog-specific behavioural alterations emerging during later developmental stages. All neurexin knockouts affected mating behaviour, complicating the generation and maintenance of homozygous lines. Except for this shared behavioural alteration, *nrxn1*, but not *nrxn2* or *nrxn3*, led to marked changes in affiliative social behaviour and aggression. In contrast, *nrxn2* mutants exhibited severe anxiety-like behaviours, including bottom-dwelling and repetitive freezing/seizure events. Strikingly, *nrxn1* full-locus deletion mutants showed opposing behaviour, spending most of their time near the surface. The two also displayed opposite responses to open/closed field transitions; confinement alleviated *nrxn2* anxiety but enhanced *nrxn1* surface-dwelling. Meanwhile, *nrxn3* mutants behaved normally in all our initial tests. In summary, our study introduces a complete set of zebrafish mutants covering the whole *nrxn* gene family, presenting striking adult behavioural alterations despite the absence of noticeable early defects; echoing the delayed onset of human psychiatric disorders such as schizophrenia. This work confirms the value of zebrafish to study mental disorders and unlock a novel platform to unravel the pathogenic contribution of neurexins and associated subtle neurodevelopmental changes/timing that drive the emergence of mental illnesses.

## Introduction

Neurexins, *NRXN1*, *NRXN2* and *NRXN3* in humans, code for transmembrane cell-adhesion molecules essential for synaptic formation, maintenance and signalling [1, 2]. These genes have repeatedly been associated with diverse mental and neurodevelopmental disorders such as Schizophrenia (SCZ), Autism (ASD), bipolar disorder (BD) and even recently with Parkinson (PD) [1–6]. A vast number of rodent models have been developed over the last two decades, which have significantly contributed to gaining tremendous insights into the biology of this complex gene family [2, 4]. Despite this tremendous effort, their pathogenic role and association with the aforementioned diseases remain unclear. Expanding the tools available to broaden our ability to interrogate these genes is still essential. Surprisingly, while the zebrafish seems to be a model of choice to tackle the function of these genes, especially in investigating their role in the developing brain, there is currently a lack of complete LOF models for research.

Indeed, complementing their rodent counterparts, the zebrafish offers a unique set of tools for studying neurodevelopment and greater versatility for drug discovery [7]. They also exhibit extra-utero development and a very convenient optical transparency, enabling direct visualisation and manipulation of their brain even during the very early developing stages. In addition, the emergence of optogenetics and calcium imaging techniques with this organism offers unique ways to strengthen the ongoing effort aiming at understanding the normal and pathogenic role of this gene family in the developing and mature brain [8, 9]. Despite these tremendous advantages, no complete LOF zebrafish neurexin models are currently available for research. This small animal presents highly conserved neurexin orthologs, which have been ancestrally duplicated -neurexin 1 (*nrxn1a* and *nrxn1b*), neurexin 2 (*nrxn2a* and *nrxn2b*) and neurexin 3 (*nrxn3a* and *nrxn3b*) [10], and although two zebrafish studies have investigated individual neurexin genes, these models target limited genomic regions using traditional small indels and therefore do not eliminate the full repertoire of neurexin isoforms [11, 12].

Indeed, these genes are transcribed into hundreds of isoforms due to multiple promoters and complex, still poorly understood, alternative splicing [13]. This complexity makes the generation of robust LOF mutations challenging. Traditional small indels -generating premature stop-codons and truncated proteins- are at high risk of being genetically compensated by de-novo and/or cryptic splicing. Let alone genetic compensation, it is not straightforward to target all isoforms at once due to the multiple promoters leading to a variety of α-long and β-short isoforms. Exemplifying these issues, the only available studies in zebrafish generated mutants affecting only the long α-isoforms, leaving the β-isoforms intact [11, 12]. Koh et al. [11] also reported that their indel triggered the use of a cryptic splice site, resulting in the removal of most of the exon carrying the mutation -confirming the presence of complex alternative splicing that complicates the study of these genes’ functions-[11]. Although aberrant splicing and compensatory mechanisms may contribute to disease pathogenesis and warrant closer investigation [14], we aimed here at first generating a set of robust zebrafish knockout lines properly missing the associated gene/protein function, and to subsequently assess their effect on zebrafish development and behaviour in order to establish a solid foundation for future mechanistic and complex genetic investigations.

Here, we are using an innovative CRISPR/Cas9 strategy to generate a complete set of zebrafish mutants covering the whole neurexin gene family and conduct a first systematic phenotyping comparison of each homozygous mutant line from embryonic to adult stage. We show that loss of these genes does not markedly affect early development or basic motor function, but results in striking and paralog-specific behavioural alterations in adulthood. Given that most mental health disorders (such as schizophrenia) emerge without detectable developmental or anatomical/structural abnormalities, while being accepted to be neurodevelopmental in origin, the presented data suggest that the zebrafish could be a valuable model to uncover pathogenic mechanisms in play prior to the symptoms. Our study also validates the zebrafish as a powerful tool to uncover the complex role of neurexins in brain development and function.

## Material and methods

### Zebrafish maintenance

Adult zebrafish and embryos were maintained by standard protocols approved by the University of Queensland and Griffith University Animal Ethics Committee. Ethics approval AE213_18/AE213_18 and GRIDD/11/22/AEC. Wildtype and all lines used in these studies are in AB background. Cryopreserved sperm from all lines presented in this study are available upon reasonable request.

### *Neurexin* mutants’ generation and maintenance

**Fig. 1:**
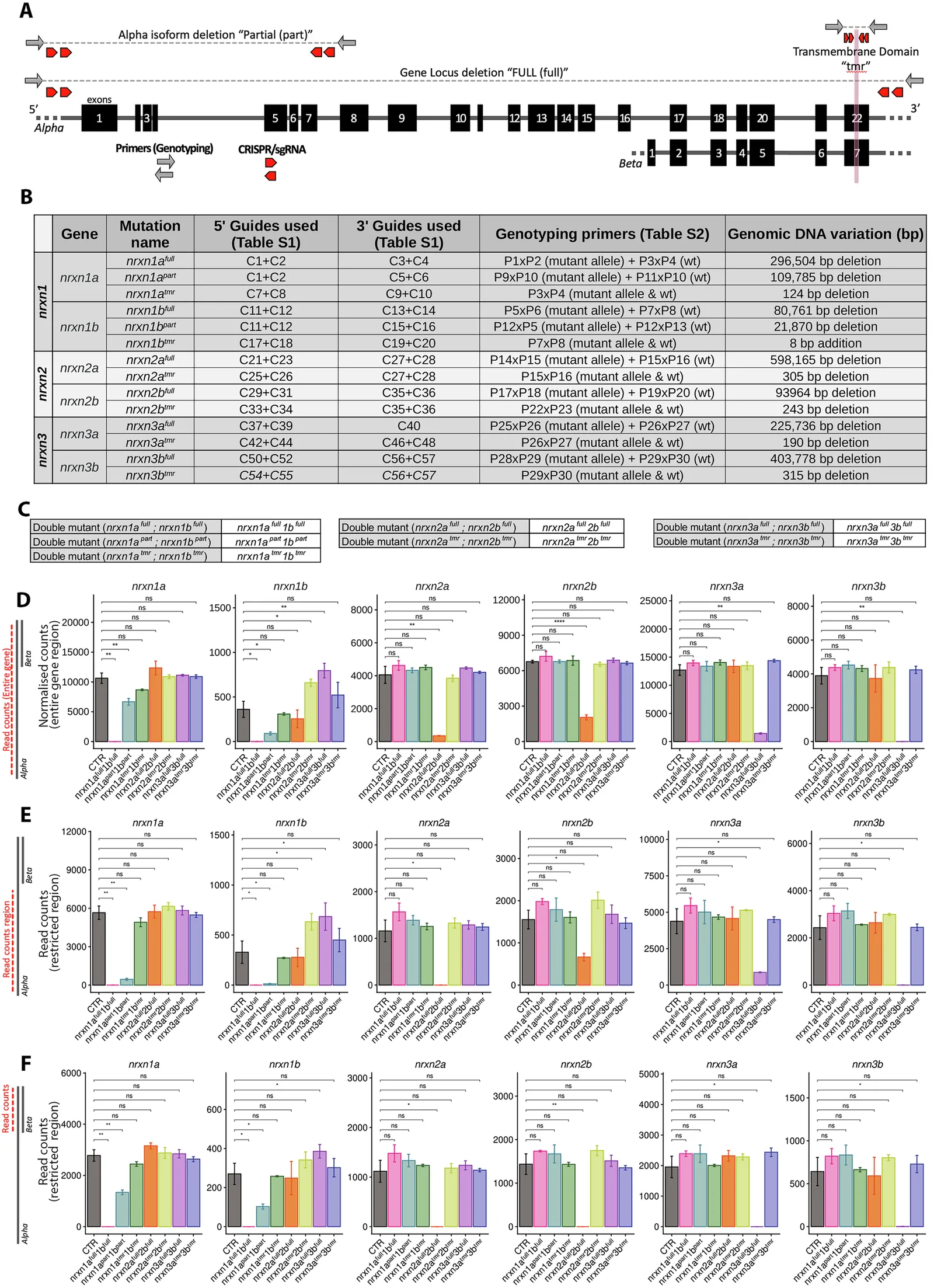
Schematic representation, details and transcriptomic consequences of the mutations generated across the six zebrafish *neurexin* genes. **A**, Schematic genomic organisation of the zebrafish neurexin loci showing the relative positions of the CRISPR/Cas9 single-guide RNAs (sgRNAs; arrowheads) used to generate the mutations/deletions described in this study. mutations/deletions presented in this study. Grey arrowheads indicate the approximate positions of the genotyping primers used for screening and maintenance of the different mutant lines. For large deletions, the identification of the homozygous mutants required two sets of primers, with the combination presented in the bottom panel. **B**, Summary of the different *neurexin* alleles/mutants generated, including corresponding i) sgRNA used (supplemental table S1), ii) genotyping primer pairs for identification and maintenance (supplemental table S2), and iii) genomic deletion sizes. **C**, Nomenclature (line names) used throughout the manuscript to designate the double mutant lines analysed in each experiment. **D-F**, RNA-seq expression analysis, with **D**, Average normalised read counts of each *neurexin* gene across condition groups, and **E-F** Average read counts for coding exons associated with the α-isoforms only (E) or shared coding exons between α- and β isoforms (F), as schematised on the left panels (genomic coordinates are specified in Supplemental Table S3). Bars represent group means; error bars show mean ± standard deviation. For each gene or region of interest, mutant lines were compared with wild type using Welch’s t-test. Complete statistical results, including *p-*values for all comparisons, are available in the Supplemental file S3.

All mutants presented in this study were generated following the protocol described in Tromp et al. [15]. CRISPR guide RNAs and targeting sequences are listed in Supplemental Table S1. Genotyping of the heterozygotes and homozygotes was performed using the primer sets presented in Fig. 1 and Supplemental Table S2. PCRs were carried out on genomic DNA extracted from dechorionated embryos or fin clips using the REDExtract-N-Amp Tissue PCR Kit (Sigma-Aldrich, Cat. XNAT-10RXN), according to the manufacturer’s instructions. Annotated sequences including sgRNA target sites, primer binding sites, and generated deletions are provided in Supplemental File S1. The homozygous analyses presented here do not include animals derived from more than two successive homozygous inbred generations. Controls and mutant animals analysed originate from the same AB genetic background.

### Embryos and larvae measurements

Embryonic and larval development were analysed using an MVX10 Microscope (Olympus, 0.63×) equipped with a DP75 digital camera and controlled with CellSens image analysis software (Olympus). Body length was measured from calibrated images, defined as the distance from the anterior tip of the head to the posterior tip of the tail. Measurements were performed on 30 larvae per genotype at 3, 5, and 7 days post-fertilisation (dpf). Hatching dynamics were assessed across multiple developmental time points at 24, 32, 40, 48, 56, 64, 72, 80, 88, and 96 h post-fertilisation (hpf). The number of hatched embryos was counted manually at each time point in multiple independent clutches for each mutant line and wild-type (wt) controls. These data were used to generate hatching curves and identify potential genotype-specific differences in developmental timing.

### Swimming-evoked response assays

Touch-evoked responses were assessed at 48 hpf in larvae from seven mutant lines and wt controls. Embryos were manually dechorionated at 24 hpf and maintained at 28.5 °C until testing. Prior to the assay, larvae were transferred to room temperature for one hour. Individual larvae (n = 30 per genotype) were placed into 90-mm Petri dishes containing E3 embryo medium. Mechanical stimuli were gently applied to the tail using a fine pestle tip. Each larva received up to ten touches, with a successful escape response scored as “1”, and absence of response “0”. Larval movements were recorded using the ZebraBox Revolution system (ViewPoint Life Sciences, France) until swimming ceased following stimulation. Swimming tracks were then analysed in FIJI (ImageJ 1.8.0_322, 64-bit), and response rates were plotted as mean ± SEM using GraphPad Prism (version 9.0.0).

### Brain imaging and brain volume quantification

To assess brain morphology, we used an established transgenic line, *Tg(HuC:Kaede)*, expressing the fluorescent protein Kaede under the control of the HuC pan-neuronal promoter, generated through Tol2-mediated genomic integration [16]. Brain scans were acquired using an Olympus FV3000 laser scanning confocal microscope. Embryos were raised in E3 medium supplemented with 0.2 mM 1-Phenyl-2-thiourea (PTU; final concentration) to block pigmentation. Larvae were scanned at defined developmental time points (33, 57, 81, 105, 129, and 153 hpf) using the 488 nm excitation channel. Acquired z-stack images were processed and analysed using FIJI (ImageJ 1.8.0_322, 64-bit) and Imaris software (v. 10.2.0 Oxford Instruments, UK). Three-dimensional brain models were reconstructed using the surface-rendering function in Imaris, applying a uniform fluorescence-intensity threshold across all samples to standardise brain-volume quantification.

### Larvae swimming and behaviour analysis

Larval swimming behaviour and response to sensory stimuli were assessed using the ZebraBox Revolution system (ViewPoint Life Sciences). Larvae were distributed in 24-well plates in triplicate, with each plate containing 12 control (AB background) and 12 mutant animals. The behavioural protocol consisted of a 32-min recording, comprising four cycles of 4 min of light and 4 min of dark. During recording, repetitive 3-s light flashes (2400 lm) and 3-s acoustic vibration (250 Hz) were delivered every minute (independently offset by 30 s). Larvae were habituated in the dark for 5 min prior to recording. Data were analysed using GraphPad Prism (version 9.0.0).

### Adult zebrafish brain dissection and RNA extraction

Adult brain dissections were completed within three days for all experimental lines. Fish were anaesthetised in an ice slurry until complete loss of movement and cessation of opercular activity were observed. Following anaesthesia, the eyes were removed, and the skulls were carefully opened using fine forceps to expose the brain. The entire brains were then excised and immediately transferred into ice-cold phosphate-buffered saline (PBS). Dissected brain samples were promptly placed into TRIzol^TM^ LS reagent (Invitrogen, California, USA) for RNA extraction. Total RNA was extracted from adult brain tissues using the Direct-zol™ RNA Miniprep Kit (Zymo Research, California, USA), which enables purification of RNA directly from TRIzol reagent without phase separation. Tissue samples were homogenised in TRIzol, and total RNA was isolated according to the manufacturer’s protocol. RNA concentration and purity were measured using a NanoDrop spectrophotometer (Thermo Fisher Scientific), and all samples were stored at −80 °C until Library preparation. Extracted RNAs were submitted for quality control (QC) assessment, library preparation, and sequencing (UQ sequencing facility, Institute for Molecular Bioscience, Brisbane, Australia). RNA integrity and concentration were evaluated prior to library construction. All RNA integrity numbers were >7.2.

### Adult zebrafish RNA sequencing and transcriptomic analysis

Samples that passed QC were processed using Illumina TruSeq Stranded mRNA Library Prep Kit. RNA sequencing was then performed using Illumina NovaSeq X platform, producing 150 bp paired-end reads (GEO accession number GSE324987). To verify the reliability of our RNA sequencing data, we performed quality control using FastQC (version 0.11.9) [17] and multiQC (version 1.9) [18]. Trimmomatic (version 0.39) [19] was implemented to remove adapters and low-quality reads. The trimmed reads were checked with FastQC and MultiQC. STAR (version 2.7.11b) was used for read alignment with the parameters “--twopassMode Basic --quantMode GeneCounts” [20]. Reads were aligned to the Ensembl zebrafish genome assembly GRCz11 (release 113) with matching GTF annotation. Expression of *neurexin* genes was normalised using DESeq2 to quantify expression per gene. Region-specific counts associated with neurexin α or β isoforms were conducted using coding exons either specific to the α isoforms only, or exons in 3’ shared between both α and β isoforms. Counts were extracted from alignment files using SAMtools [21], with genomic coordinates for these regions provided in Supplementary Table S3.

### Adult behavioural studies

All adult behavioural experiments (described below) were performed using Zantiks LT automatic system (Zantiks Ltd., Cambridge, UK; scripts used in this study are available in Supplementary File S2). All the tests were conducted between 11 am and 6 pm to minimise circadian disruption. The experimental room was maintained under constant temperature and illumination conditions. Mutant lines were tested in parallel with wt controls and comparisons were restricted to batches performed within the same timeframe. Fish were used with an approximately balanced sex ratio (male:female ~ 1:1) in each group. All adult behavioural experiments were conducted with animals between four and six months of age.

### Novel tank diving test

The Novel Tank Diving test (also known as the Novel Tank Test, NTT) uses vertical distribution of zebrafish in a novel environment as a validated measure of ‘anxiety-like’ behaviours in adult zebrafish [22], and was performed following established protocols [23]. Fish were acclimatised for at least 1 h in the experimental room prior to behavioural testing. Each zebrafish was placed in a transparent tank of 125 × 155 (mm) dimensions (T-225 cell culture flask, supplied by Zantiks Ltd., Cambridge, UK). Fish behaviours were recorded for 15 min in lateral view and tracked via the Zantiks Software interface. For data analysis, the tank was virtually divided into top (T), middle (M), and bottom (B) zones. Total distance travelled, average time spent in each zone, and average entries into each zone of the tank were generated in real time using the Zantiks in-built software. Average velocity, latency to first entry into the top zone, number of freezing bouts and freezing percentage were subsequently quantified using EthoVision XT software (Ver. 18.0.1803, Noldus, Netherlands).

### Mirror biting test

The mirror biting is a well-established fish paradigm [24, 25]. The test was conducted in opaque tanks (200 × 140 × 140 mm) to minimise visual distractions from the external environment. A mirror insert was placed along one side of the tank, with two mirrors fitted at an angle to enhance the reflective surface [26]. The mirror zone was defined as the area within 3 cm of the mirror. Each fish was individually acclimated in the tank for 5 min in the dark. Following acclimation, illumination was restored, exposing the fish to the mirrors, and behaviour was recorded for 10 min. The total time spent within the mirror zone was calculated as the primary measure of mirror-directed behaviour.

### Social preference test

The social preference assay was designed to evaluate social interaction and preference in zebrafish [25, 27]. An individual test fish was introduced into the central compartment of an opaque testing tank, with a group of same-strain conspecifics placed in an adjacent compartment at one end. The opposite end contained an empty compartment, serving as a neutral control. The central compartment was further virtually divided into 4 equal-sized subzones; the zone nearest to the friend fish was designated “social zone”, the 2 zones in the middle were designated “middle zone”, and the third zone was designated “empty zone”. Behavioural recordings started after an acclimation of 5 min. Videos were recorded from a top view of the tank for 10 min. The time spent in each zone was then calculated and the social preference index was computed to quantify the degree of social interaction exhibited by the test fish.

### Closed field test

The principle of this assay is similar to the Novel Tank Test (NTT); however, it has been adapted in our lab to assess anxiety-like behaviour in a group of fish rather than in individuals. This assay is referred to as the Closed Field Test, as it was conducted within a confined environment inside the Zantiks LT chamber, surrounded by black foam panels to cut off any external visual cues, providing a sheltered environment. This contrasts with the Open Field Test (described below), where the tank is placed in an open environment external to the Zantiks system, leaving the animal exposed to the surrounding space (illuminated and with a large field of view). The Closed Field Test was performed in a transparent rectangular tank (370 × 140 × 300, mm, length × width × height). A group of 10 fish was gently placed into the tank, and their behaviour was recorded from lateral view. During the experiment, the integrated overhead LED illumination of the Zantiks system was turned on and maintained at a constant intensity across all trials. The shoal was recorded for 15 min, including a 5-min acclimatisation and a 10-min experimental recording. The experiment was repeated four times (n = 4 independent groups of 10 fish; 40 fish in total). The recorded videos were imported into Adobe Premiere Pro 2022 (Adobe Inc., California, USA) using a custom overlay grid consisting of three horizontal lines that divided the tank height into three equal zones (defined as top (T), middle (M), and bottom (B)). The number of fish in each zone was manually counted every 5 s. Shoaling distribution was scored blind to genotype.

### Open field test

This assay was performed in the same experimental room and used the same rectangular tank as in the Closed Field Test but installed in an open setting outside the Zantiks LT chamber. The tank was positioned on a table placed in the centre of the 3 × 3 m experimental room, and illuminated by constant ceiling lights (Australian standard lighting, 400 lux at 700 mm AFFL as per AS/NZS1680.2.4). To ensure consistency, water depth was maintained at the same level as in the Closed Field Test. A light panel (x-ray viewer panel, Edwards instruments, NSW), was positioned 40 cm behind the tank to improve contrast and animal detection. Behavioural recordings were captured from the side using a Logitech Brio 4 K Pro Webcam (Logitech International S.A., Lausanne, Switzerland) connected to the Zantiks Console control system. As in the Closed Field Test, each 10-fish group was gently netted into the tank, and their behaviour were recorded for 15 min (5 min acclimatisation), following the same analytical pipeline. Four groups (n = 4 independent groups of 10 fish; 40 fish in total) were tested per genotype. Shoaling distribution was scored blind to genotype.

### Swimming tunnel assay

This test was conducted using a swimming tunnel apparatus from Loligo® Systems (Madison, USA), with protocol modifications based on a previous study [28]. A group of 4 fish was placed into the glass chamber, and behaviour was recorded laterally using a “Logitech BRIO – Ultra HD Webcam”. Animals were first acclimated for 5 min in static water. Following acclimation, water velocity was increased stepwise by 10 cm/s every 5 min to induce flow-driven swimming behaviour. The time from the onset of flow to exhaustion was recorded, with exhaustion defined as the point at which the fish could no longer maintain position within the flow and were carried onto the collection mesh at the downstream end of the chamber. The moment a fish ceased active swimming was recorded as its time to exhaustion.

### PTZ treatment experiment

Pentelenetetrazole (PTZ; P6500-25G) was purchased from Sigma-Aldrich (Saint Louis, MO, USA). A 2 M stock solution was prepared in UltraPure™ DNase/RNase-Free Distilled Water (Invitrogen, USA) and stored at −20 °C until use. For the treatment, adult wt and *nrxn* mutant fish were acclimatised in the experimental room for at least 1 h before the test. PTZ final concentration (0.1, 1, 5 and 10 mM) and exposure time were chosen based on previous studies [29, 30]. The control group was exposed to system water only. Working PTZ solutions were freshly prepared by diluting the stock solution in 250 mL of system water and added to the experimental tanks (same tanks as the ones used in the novel tank diving assay). Individual fish were gently netted into the tanks, and their behavioural activity was recorded for single-20 min sessions. Following the experiment, fish were transferred to recovery tanks containing fresh system water. Fish health and survival were monitored for one week before the animals were returned to their home housing tanks. Treatment water was changed after each session. Seizure scoring was performed according to previously published criteria [30].

### Statistical analysis

Sample size was determined by the number of animals available for each genotype, while maintaining sex balance, and is consistent with prior studies using similar behavioural paradigms, where such group sizes are sufficient to detect robust phenotypic differences [22, 27, 28]. Statistical analysis was performed using GraphPad Prism 9 software (GraphPad, La Jolla, CA, USA) and R (version 4.5.2). No data were excluded. No randomisation was used. Investigators were not blinded during the experiments, except where stated otherwise. Data are presented as mean ± SEM. Comparisons between groups were conducted using statistical tests indicated in the figure legends. In the figures, * indicates p < 0.05, ** indicates p < 0.01, *** indicates p < 0.001, and ns indicates no significance. A detailed summary of all statistical tests and results is provided in Supplemental File S3.

## Results

### Zebrafish neurexin (*nrxn*) mutants’ generation

To generate LOF zebrafish *nrxn* mutants, we employed a CRISPR/Cas9 methodology previously established in our laboratory to facilitate the generation of targeted large deletions across the genome [15]. We applied this approach to generate mutants for all zebrafish *nrxn* genes, *e.g. nrxn1a*, *nrxn1b*, *nrxn2a*, *nrxn2b*, *nrxn3a*, and *nrxn3b*, and established double-mutant colonies for phenotypic investigations. We slightly modified the published technique/methodology to use only four guides (*e.g*. minus the described fifth guide targeting the gene tyrosinase as a selection marker); with two guides on each side of the deletion of interest as described in Fig. 1A. To avoid genetic compensation via alternative splicing as well as enable the study of non-coding regions across these genome loci, we aimed to remove i) the entire genomic regions, including all introns (deletions named “full” in this manuscript) or ii) each transmembrane region (located on the last exon of each gene; deletions named “tmr”). For *nrxn1*, we also generated an additional line with a deletion/mutation running from the α-isoform promoter to Exon 7 (named “part”), which does not overlap with the genomic region associated with the short *nrxn1*-isoforms, *nrxn1a-β* and *nrxn1b-β*. The details of these deletions are presented in Fig. 1B, with the corresponding annotated sequences provided in Supplemental File S1, and all guide RNAs and genotyping primers listed in Supplemental Table S1 and Supplemental Table S2. Following the selection/isolation of these different mutations, we worked at establishing stable colonies to study each neurexin independently in a double mutant context, named here *nrnx1a1b*, *nrxn2a2b* and *nrxn3a3b* (summarised in Fig. 1C). As presented below, all these mutants and double mutants are viable. The sperm of all generated single and double mutants have been cryopreserved and are available on demand. RNA-seq analysis confirmed the expected molecular consequences of these mutations (Fig. 1D-F, Supplemental File S3). Complete genomic deletions resulted in a strong reduction or absence of the corresponding transcripts, whereas transmembrane-domain mutations did not affect transcript levels, consistent with their design to disrupt protein localisation without altering gene expression. Similarly, none of the mutations triggered evidence of transcriptomic compensation among neurexin paralogs, with the exception of a slight increase in *nrxn1b* expression in double mutants *nrxn2a*^*tmr*^*2b*^*tmr*^ and *nrxn3a*^*full*^*3b*^*full*^, which should be interpreted with caution due to its very low baseline expression relative to other neurexins.

### Neurexins have no gross impact on the early development of zebrafish embryos and larvae

**Fig. 2:**
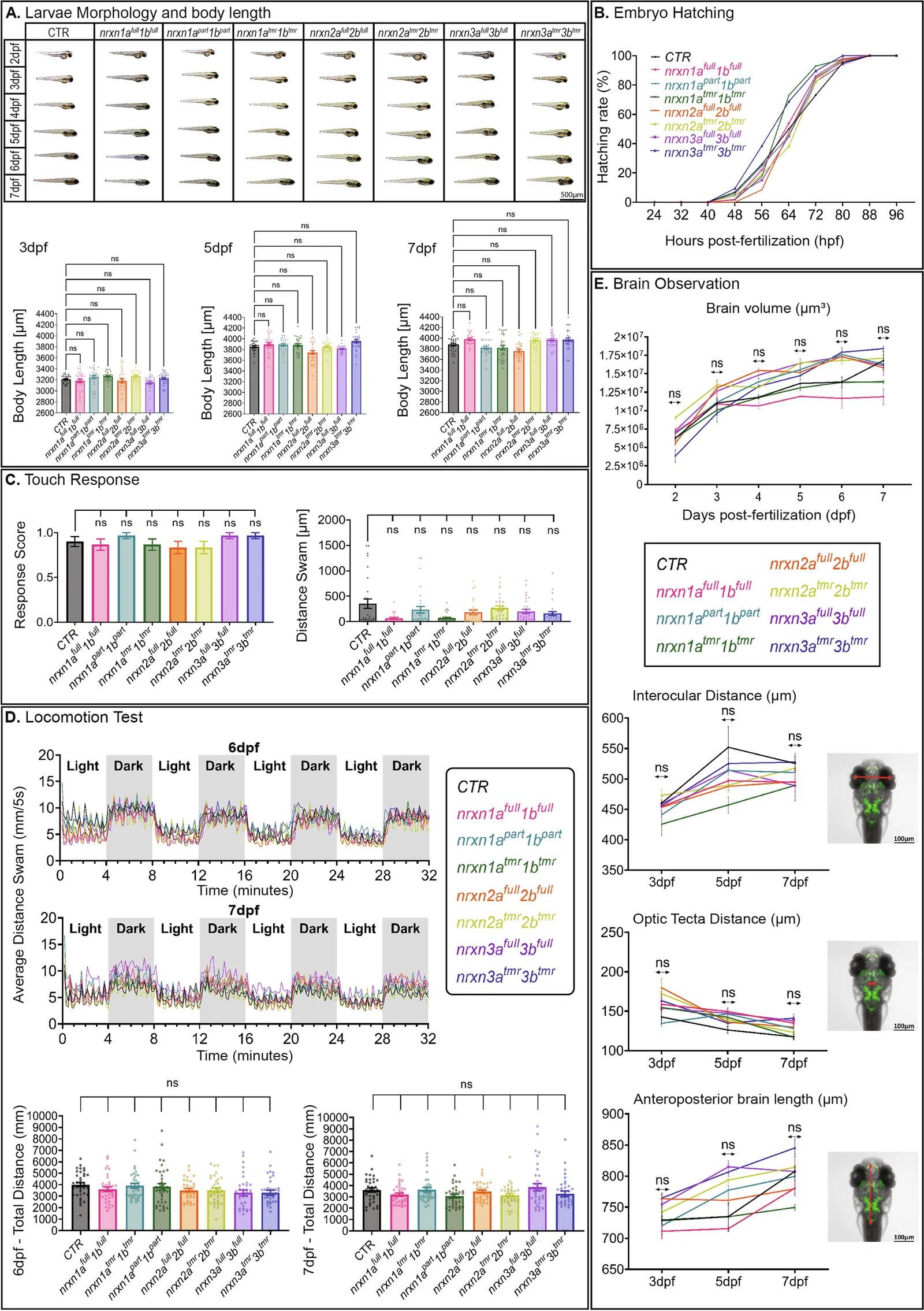
*nrxn1*, *nrxn2* and *nrxn3* are not essential to zebrafish gross development and early swimming behaviour. **A**, Upper panel, representative lateral view of the different zebrafish *neurexin* mutants versus wild-type control from 2 dpf to 7 dpf. Lower panel, body length measurement at 3, 5 and 7 dpf demonstrated no difference to wild-type control (n = 30 per group); Scale bar = 500 µm. **B**, Hatching rates (%) of *neurexin* mutants versus wild-type larvae from 24hpf to 96hpf (n ≈ 300 per group). **C**, Touch-evoked response scores (left panel) and swimming response (right panel) of the different neurexin mutants vs wild-type control (n = 30 per group). **D**, Swimming behaviour analysis and average distance travelled during a 32-min recording, including alternating light/dark stimulation and sound/vibration stimuli; test conducted at both 6 and 7dpf. Individual tracks are shown in Supplemental Fig. S1. The total distance travelled across the experiment demonstrated no statistical difference across all lines tested (n = 36 per group). **E**, Real-time brain development and volume analysis demonstrated no statistical difference between groups. Brain volume (upper panel) was monitored from 2 to 7 dpf, and the distance between brain landmarks (interocular/optic tectum/brainstem) was quantified at 3, 5, and 7 dpf (lower panels); (n = 3 per genotype). Scale bar = 100 µm. Comparisons among all mutant lines and the control were performed using the Kruskal-Wallis test, followed by Dunn’s multiple comparison test. (*ns*, no significance).

**Fig. 3:**
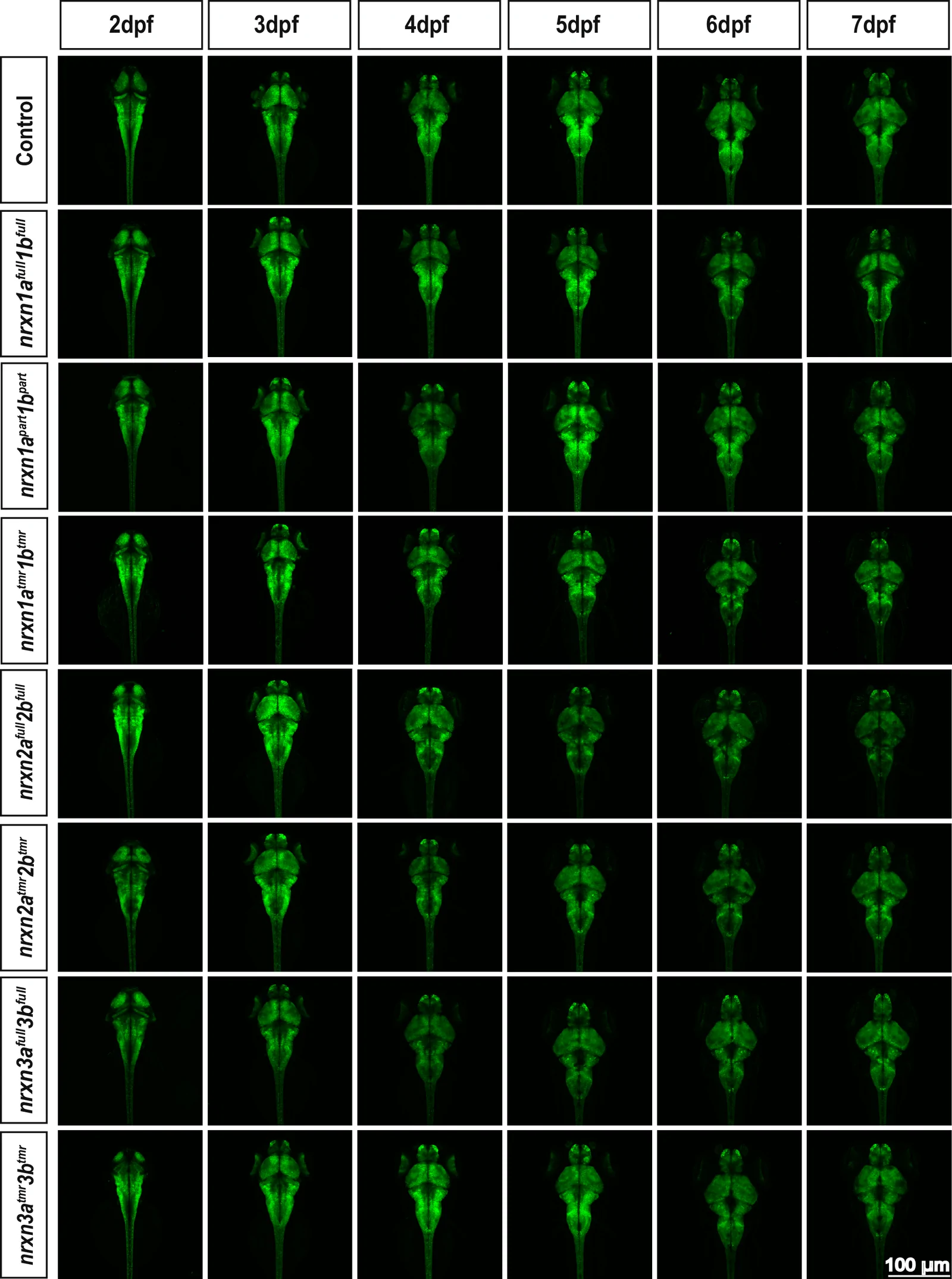
*nrxn1*, *nrxn2* and *nrxn3* are not essential for gross brain and nervous system development in zebrafish. Confocal brain scans of transgenic control (CTR ; *tg(huc:Kaede)*) versus the different *neurexin* mutants in the transgenic *tg(huc:Kaede)* background. The transgene *tg(huc:Kaede)* drives expression of the fluorescent protein KAEDE in the whole nervous system. Confocal micrographs (max intensity z-stack projections) show dorsal views of whole brains acquired daily from 2 to 7 dpf, illustrating progressive brain development and the absence of overt structural abnormalities across genotypes. Scale bar = 100 µm.

Following the successful generation of double-mutant lines, we conducted comparative phenotypic analyses. Unexpectedly, at embryonic and larval stages, none of the mutants displayed overt phenotypes in our initial comparative tests. Mutant animals hatched and developed without significant differences to their wt control counterparts (Fig. 2A and B). Control wt animals grew from 3209 µm ( ± 66.86 µm) at 3 days-post-fertilization (dpf) to 3877 µm ± 136.1 µm at 7 dpf with no statistical differences between groups at any time point (Fig. 2A, detailed statistics available in Supplementary File S3). All clutches tested were fully hatched by 3 dpf and grew with no observable morphological defects (Fig. 2B). At 2 dpf, all animals/mutants responded to touch with no difference in their swimming response/patterns compared to controls, suggesting no detectable delay or defect in brain or peripheral nervous system development at embryonic stage (Fig. 2C). We further analysed their behaviour up to 7 dpf using the Zebrabox Revolution platform and a battery of stimuli (Fig. 2D and Supplemental Fig. S1). None of our experiments evidenced a significant change in their spontaneous behaviour or response to stimuli. Figure 2D summarises behavioural profiles of all seven double-mutant lines exposed to a 24-min protocol combining light–dark transitions, vibration/sound, and illumination stressors. All mutants exhibited swimming patterns and response intensities comparable to controls, indicating no early obvious changes in behaviour or neural function. Finally, we monitored brain development from 2 to 7 dpf and no differences in overall structure, size, or developmental timing were observed (Figs. 2E and 3), supporting the conclusion that embryonic to larval nervous system development proceeds essentially normally in the absence of neurexins.

### Absence of obvious early defects translated into profound adult behavioural alterations

**Fig. 4:**
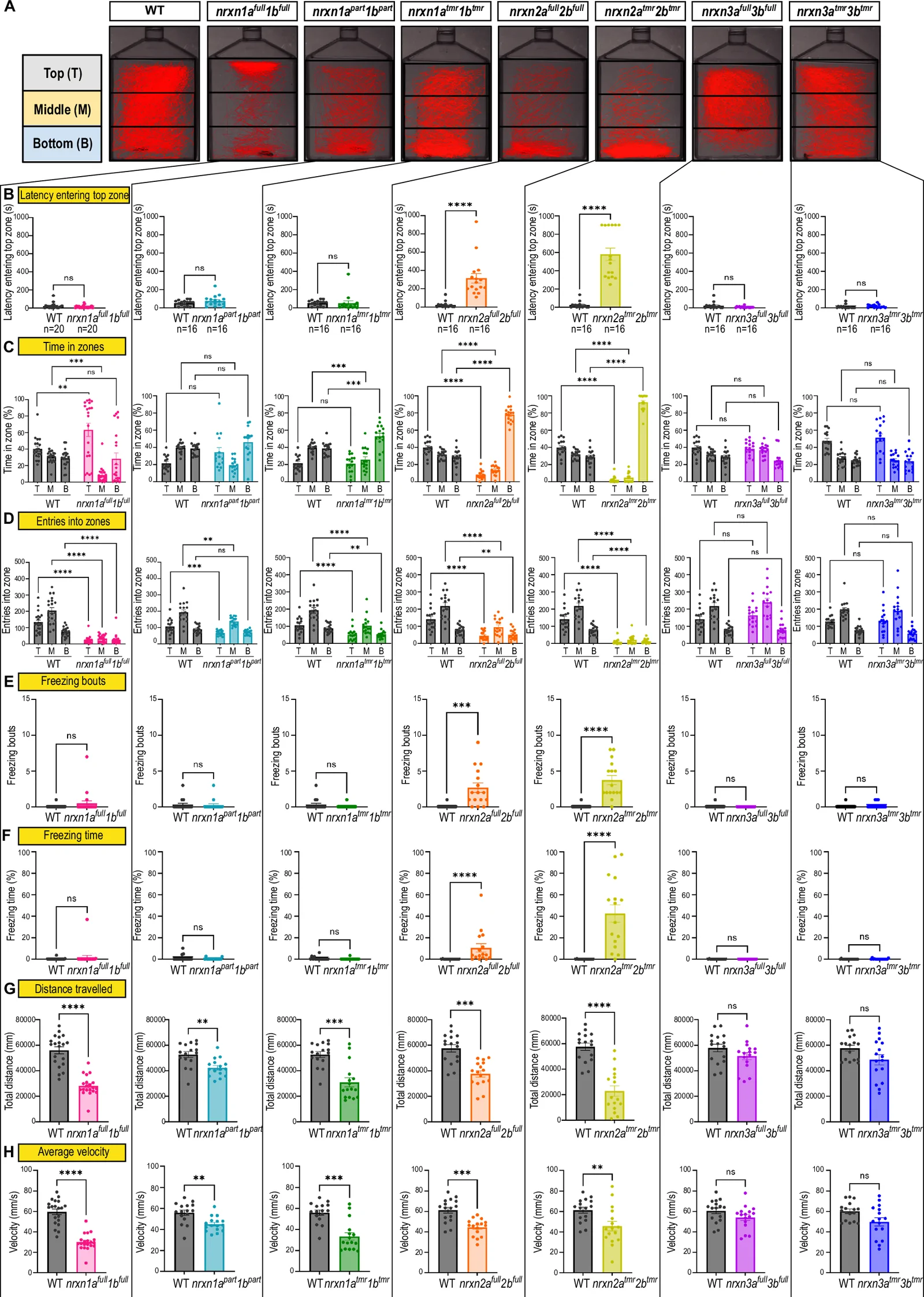
Neurexins dramatically affect adult zebrafish behaviour, with paralog-specific alterations. Novel Tank Diving Tests demonstrated that *nrxn2* deletion triggers robust anxiety-like phenotype characterised by persistent bottom-dwelling swimming coupled with repetitive freezing, whereas *nrxn1* full locus removal promotes atypical surface-swimming behaviour. **A**, Schematic diagram of the novel tank diving test and representative swimming tracks of wild-type versus the different *neurexin* mutant lines (15-min recordings). **B**, Latency to first entry into the top zone. **C**, Total time spent in top, middle, and bottom zones of the tank during the 15-min recordings. **D**, Cumulative number of entries into each zone. **E**, Percentage of time spent frozen during the 15-min recording. **F**, Cumulative number of freezing bouts. **G**, Total distance travelled during the test. **H**, Average velocity during mobile periods (freezing periods/time excluded). Data are presented as mean ± SEM. For comparisons between two groups in panel B, E, F, G, H, two-tailed Mann-Whitney tests were used, and *p*-values were subsequently corrected across the seven mutant lines using false discovery rate (FDR) correction. Comparisons between genotypes across the three zones in panel C (Time in zone, %) were analysed using aligned rank transform ANOVA (ART ANOVA). Post-hoc pairwise comparisons between wild-type and mutant fish within each zone were performed using the “*emmeans”* package in R. For panel D (Entries into zone), which represents count data, a generalized linear mixed model (GLMM) was used, followed by post-hoc pairwise comparisons. The *p*-values obtained from post-hoc comparisons across the seven mutant lines for a given behavioral endpoint were pooled and adjusted simultaneously using FDR correction. (* indicates p < 0.05, ** indicates p < 0.01, *** indicates p < 0.001, **** indicates p < 0.0001).

**Fig. 5:**
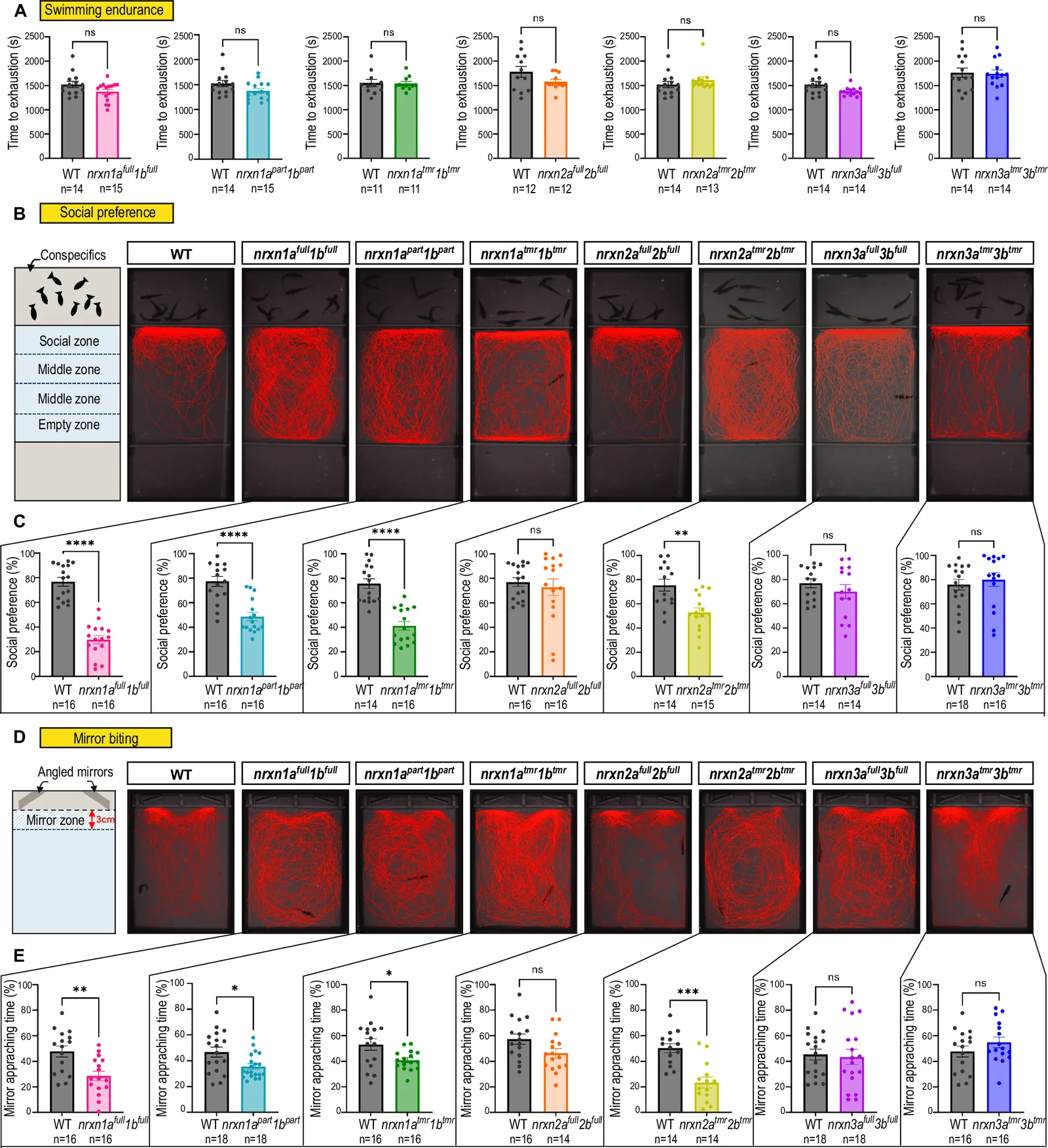
*nrxn1* modulates affiliative social behaviour and aggression in zebrafish. **A**, Swimming endurance assays, with performance expressed as time to exhaustion. **B-C**, Social preference assays with: **B**, Schematic diagram of the social preference test/apparatus, and representative swimming tracks of wild-type versus the seven *neurexin* mutant lines during 10-min recordings. **C**, Quantification of the percentage of time the tested fish spent in the social zone. **D-E**, Mirror biting assays with: **D**, Schematic diagram of the mirror biting test/apparatus and representative swimming tracks of wild-type versus the different *neurexin* lines during the 10-min test. **E**, Percentage of time the fish spent in the mirror zone. Data are shown as mean ± SEM. For comparisons between wild-type and mutant, two-tailed Mann-Whitney tests were used. False discovery rate (FDR) correction was applied to control for multiple testing across the seven mutant lines in each behavioral endpoint (* indicates p < 0.05, ** indicates p < 0.01, *** indicates p < 0.001, **** indicates p < 0.0001).

**Fig. 6:**
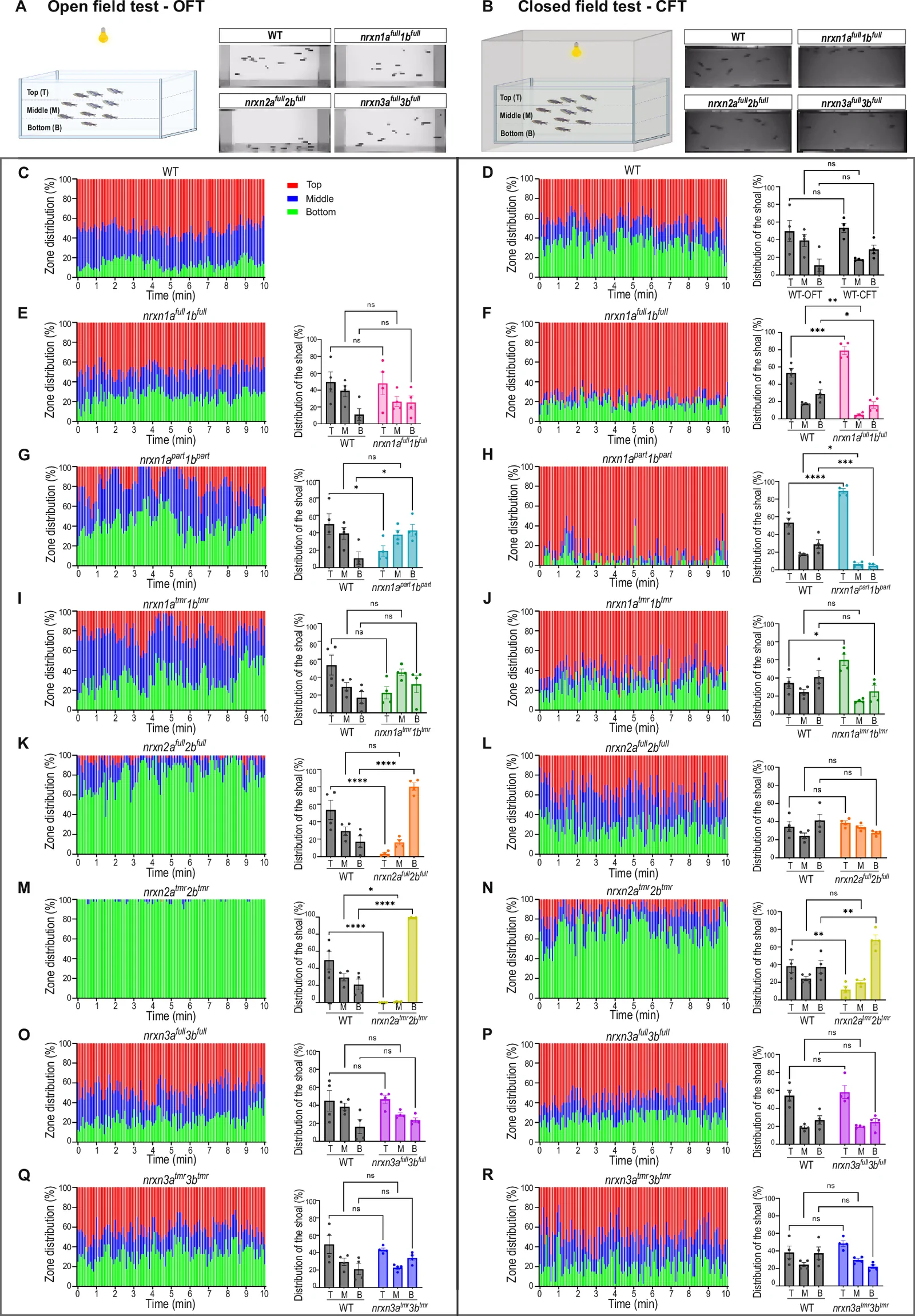
*nrxn1* and *nrxn2* mutant lines display a divergent response to open and closed field environments. **A**, Schematic representation of the Open Field Test apparatus and video-snapshots examples. **B**, Schematic representation of the Closed Field Test apparatus and video-snapshots examples. **C-R**, Zone preference over time of wild-type and the seven studied neurexin mutants. **Left panels** represent the average number of fish in each zone every 5 s through the 10 min recordings (Percentage of 10 fish per 5 s, with 4 independent groups; n = 4 with 40 fish total). **Right Panels** average the overall spatial distribution of the 10 recorded fish over the total experimental recording of 10 min (Percentage of 10 fish over 10 min recordings, with 4 independent groups; n = 4 with 40 fish total). Experiments started after 5 min of habituation. Data are shown as mean ± SEM. For comparisons between two groups across three zones, ART ANOVA was applied. Post-hoc pairwise comparisons between wild-type and mutant fish within each zone were performed. All raw *p*-values from post-hoc comparisons were simultaneously adjusted using the Benjamini-Hochberg false discovery rate (FDR) procedure to control for multiple testing across the seven mutant lines within each behavioural condition (* indicates *p* < 0.05, ** indicates *p* < 0.01, *** indicates *p* < 0.001, **** indicates *p* < 0.0001).

While no obvious behavioural or developmental defects were detected at early stages, pronounced behavioural alterations emerged later in life. While all mutations affected mating behaviour, rendering the maintenance and generation of embryos challenging, we observed clear paralog-specific phenotypes. This was first evident in the facility per se., where *nrxn2a*^*full*^*2b*^*full*^ and *nrxn2a*^*tmr*^*2b*^*tmr*^ mutants displayed a nearly permanent bottom-dwelling behaviour, spending all day at the bottom of the tanks, even during feeding routines (Supplemental Video S1). These striking differences prompted a systematic behavioural assessment using a battery of tests, including novel tank diving, swimming endurance, social preference, mirror-biting, and shoaling assays (open- versus closed-field), presented below and in Figs. 4 to 6.

### Loss of *nrxn2* triggers profound anxiety-like behaviour

Following the observed consistent bottom-dwelling behaviour of *nrxn2a*^*full*^*2b*^*full*^ and *nrxn2a*^*tmr*^*2b*^*tmr*^ mutants within the facility, we subjected them to a novel tank diving test, a well-established assay for assessing anxiety-like behaviour in zebrafish [31]. In this paradigm, fish are introduced into a novel environment, and the latency to reach the upper section of the tank is considered inversely correlated with anxiety -*e.g*. the longer the animal stays at the bottom, the higher the anxiety level. Wild-type controls reached the top section within an average of 23.16 ± 8.8 s, whereas *nrxn2a*^*full*^*2b*^*full*^ and *nrxn2a*^*tmr*^*2b*^*tmr*^mutants exhibited significantly prolonged latencies, with an average time to reach the top section of 314.4 ± 8.8 s (Mann-Whitney test, *U* = *1*, *p* < 0.0001) and 580.1 ± 66.8 s (*U* = *0*, *p* < 0.0001), respectively (Fig. 4A-B). Strikingly, 37.5% of *nrxn2a2b*^*tmr*^ mutants never explored up to the upper section, remaining exclusively at the bottom throughout the assay, indicating a severe anxiety-like state in zebrafish.

Consistent with these findings, analysis of vertical distribution and number of transitions between the three zones revealed a marked reduction of their exploratory behaviour when compared to controls (Fig. 4C-D). Regarding vertical distribution, the statistical analysis confirmed a pronounced change in behaviour (ART ANOVA, genotype x zone interaction: *nrxn2a*^*full*^*2b*^*full*^, *F*(2,60) = 287.35, *p* < 0.0001; *nrxn2a*^*tmr*^*2b*^*tmr*^, *F*(2,60) = 347.12, *p* < 0.0001); while wt fish spent a cumulative 28.6 ± 2.3% of the assay in the lower section, *nrxn2a*^*full*^*2b*^*full*^ and *nrxn2a*^*tmr*^*2b*^*tmr*^ mutants remained almost exclusively at the bottom, spending 78 ± 2.4% and 92 ± 2.4% of the total experimental time in the lower section, respectively (post hoc p < 0.0001, Fig. 4C). Demonstrating not only a bottom-dwelling but also a clear reduction of their explorative behaviours, while wt fish performed 421 ± 45 cumulative transitions between sections during the assay, *nrxn2a*^*full*^*2b*^*full*^ mutants made only 192 ± 25 transitions (Mann-Whitney test, *U* = 36.5, *p* = 0.0003), and *nrxn2a*^*tmr*^*2b*^*tmr*^ mutants rarely exited the bottom zone, with only 43 ± 15 total transitions across the trial (*U* = 2, *p* < 0.0001) (Fig. 4D).

### Loss of *nrxn2* triggers robust and repetitive freezing/seizures, but does not affect swimming performance

In addition to a profound bottom-dwelling and reduction of exploration, *nrxn2a*^*full*^*2b*^*full*^ and *nrxn2a*^*tmr*^*2b*^*tmr*^ also displayed repetitive and robust freezing behaviours often followed by a burst of erratic hyperactivity/swimming, observed both during the behavioural assays and during routine observations in the facility (Fig. 4E-F, Supplemental Video S2/S3). In the novel tank diving tests, *nrxn2a*^*tmr*^*2b*^*tmr*^ displayed freezing behaviour for a mean of 43 ± 8% of the experimental time, significantly higher compared to wt (Mann-Whitney test, *U* = 8.5, *p* < 0.0001). In comparison, *nrxn2a*^*full*^*2b*^*full*^ froze for 11 ± 4% of the experimental time, appearing less affected than *nrxn2a*^*tmr*^*2b*^*tmr*^ (*U* = 25.5, *p* < 0.0001). Further characterisation of these freezing episodes during the novel tank diving tests with the *nrxn2a*^*tmr*^*2b*^*tmr*^ mutants revealed the presence of two types of events, periods of freezing followed by normal return to swimming activity (with animals often lying at the bottom of the tank) and freezing episodes immediately followed by abrupt bursts of erratic hyperactivity. Quantitative analysis of the *nrxn2a*^*tmr*^*2b*^*tmr*^ novel tank diving recordings showed an average of 4 ± 0.6 freezing events per assay, with 20.8 ± 7.7% of these episodes immediately followed by high-velocity burst of erratic activity (Fig. S3A-C, Supplemental Video S3). Such freezing-burst sequences are reminiscent of seizure-like behaviours described in zebrafish models of network hyperexcitability [30, 32]. To further probe network excitability pharmacologically, we exposed fish to the GABA_*A*_ receptor antagonist pentylenetetrazol (PTZ). *nrxn2a*^*tmr*^*2b*^*tmr*^ displayed marked hypersensitivity to PTZ, showing significantly increased susceptibility compared with wt controls when exposed to 1 mM PTZ (Fig. S3D-G). Although electrophysiology and/or calcium imaging experiments would be required to formally confirm the epileptiform nature of these events, these results are consistent with increased network excitability and seizure susceptibility. In addition, *nrxn2a2b* mutants, along with all *nrxn1a1b* but not *nrxn3a3b* mutants, showed a marked reduction in total distance travelled and average swimming velocity during these novel tank diving experiments (Fig. 4G and H**)**. These values were calculated after excluding freezing periods to avoid bias. To determine whether these reductions reflected neuromuscular/fitness impairment rather than altered behaviour, the different lines were subjected to an endurance assay using a Loligo® swimming tunnel, in which the animals were challenged with a progressively increasing water flow. As presented in Fig. 5A, mutants and wt animals displayed comparable resistance to increasing flow, indicating preserved strength and endurance, suggesting that the observed reduced distance swam and velocity arise primarily from behavioural alterations rather than motor deficits.

### Loss of *nrxn1* triggers unusual surface-dwelling behaviour coupled with reduced exploration

In contrast to *nrxn2a2b*, *nrxn1a1b* mutants did not exhibit strong anxiety phenotypes, but instead demonstrate marked defects/changes in their affiliative social behaviours and aggression (see below and Fig. 5). Before describing these new sets of assays/phenotypes, it is noteworthy to highlight that, during the novel diving tests, none of the *nrxn1a1b* mutants demonstrated significant change in their latency to enter the top zone (Fig. 4A and B – concluding on an absence of significant anxiety). However, *nrxn1a*^*full*^*1b*^*full*^, specifically, demonstrated a pronouncedly significant top-section swimming behaviour/preference (Fig. 4C), combined with less exploration/transition to the middle and bottom zones and less overall distance travelled (Fig. 4D, G and H, Supplemental Video S2). To our knowledge, this combination of marked top section preference coupled with reduced exploration and reduced distance-swam/velocity has not been previously described and suggests profound and potentially complex behavioural alteration. Interestingly, while *nrxn1a*^*part*^*1b*^*part*^ and *nrxn1a*^*tmr*^*1b*^*tmr*^ did not share this significant top section preference in the novel tank diving test, they demonstrated a similar significant surface-dwelling behaviour in the closed field tests presented below (Fig. 6).

### Loss of *nrxn1* modulates social preference and aggression

In addition to their surface-dwelling behaviour, all *nrxn1a1b* mutants developed marked changes in affiliative social behaviour and aggression (Fig. 5B-E, Supplemental Video S4 and Supplemental Video S4). To assess social preference, we used three-chambered experimental tanks in which two small side compartments were separated from the central arena by transparent glass. One side compartment contained a group of conspecifics, while the opposite compartment was left empty. The test fish was placed in the central arena, and its spatial distribution was analysed to quantify social preference/behaviour. As shown in Fig. 5B and C, while wt control spent 77 ± 4% of the experimental time adjacent to their sibling, *nrxn1a*^*full*^*1b*^*full*^, *nrxn1a*^*part*^*1b*^*part*^ and *nrxn1a*^*tmr*^*1b*^*tmr*^ mutants exhibited a pronounced social interaction withdrawal, spending only 30 ± 3.4% (Mann-Whitney test, *U* = 0, *p* < 0.0001), 49 ± 3.5% (*U* = 25, *p* < 0.0001) and 43 ± 3.8% (*U* = 12, *p* < 0.0001) of the experimental time, respectively, near conspecifics. To assess aggression, we deployed a mirror biting test (Fig. 5D and E). Wild-type control animals spent an average of 48 ± 3.7% within the 3 cm zone next to the mirror. In contrast, all *nrxn1a1b* mutants demonstrated a significant reduction of their time interacting with the mirror, with 29 ± 3.7% for *nrxn1a*^*full*^*1b*^*fulll*^ (Mann-Whitney test, *U* = 50, *p* = 0.0046), 36 ± 2% for *nrxn1a*^*part*^*1b*^*part*^ (*U* = 84, *p* = 0.0226) and 40 ± 1.9% for *nrxn1a*^*tmr*^*1b*^*tmr*^ (*U* = 66, *p* = 0.0265). To further support these results, the *nrxn1a*^*full*^*1b*^*fulll*^ mutants were further subjected to a dyadic fighting test [33]. As presented in Supplemental Fig. S4, the mirror-biting findings were corroborated by a robust decrease in direct aggressive interactions, with mutants displaying only 2 ± 1 bites per minute on average, compared with 16 ± 3 bites per minute in the wt controls (*U* = 0, *p* = 0.0286). It is noteworthy that the mirror biting tests also concluded on a significant alteration of social preference and aggression for *nrxn2a*^*tmr*^*2b*^*tmr*^ animals; however, the high frequency of freezing and seizure-like episodes observed during the tests may have confounded these results, and the significance of these findings should therefore be interpreted with caution.

### *nrxn1* and *nrxn2* mutants display opposite responses to open and closed field tests

Building on the initial behavioural analyses conducted with single animals, we next assessed group behaviour by analysing shoal distribution in an open versus closed field apparatus. The main goal was to confirm an observation made in the facility during daily maintenance; *nrxn2a2b* mutants, typically exhibiting persistent bottom-dwelling, began to explore their tanks when placed in a protected or covered area. This contrasted sharply with their constant bottom-dwelling behaviour when tanks were positioned on the open workbench or openly exposed in the facility racks. For the experiment, the same test tanks were used in both open- and closed-field conditions; the key distinction was that in the closed-field setup, the tank was fully covered, providing shelter and shielding the fish from external cues (Fig. 6B). In contrast, in the open-field setup, the tank remained uncovered, leaving animals fully exposed to their surroundings. Groups of ten fish were introduced in the testing tank, and fish distribution was analysed over time and plotted in Fig. 6. Replicating the observations made in the facility and during the novel tank diving test, all *nrxn2a2b* mutants in the open field apparatus displayed a robust and persistent bottom-dwelling behaviour, with an average of 80 ± 4% and 99 ± 0.3% of the shoal of *nrxn2a*^*full*^*2b*^*full*^ and *nrxn2a*^*tmr*^*2b*^*tmr*^ distributed at the bottom of the tanks across the entire duration of the experiment; compared with only 17 ± 6% in wt controls (Fig. 6K-N). This difference was highly significant (ART ANOVA, genotype x zone interaction: *nrxn2a*^*full*^*2b*^*full*^, *F*(2,12) = 59.071, *p* < 0.0001; *nrxn2a*^*tmr*^*2b*^*tmr*^, *F*(2,12) = 99.989, *p* < 0.0001), with post-hoc comparisons confirming increased bottom occupancy in both mutant lines (*p* < 0.0001). Confirming our observation, when transferred to closed-field conditions, *nrxn2a*^*full*^*2b*^*full*^ did not behave differently from wt, with the shoal distribution in each zone being the same as in wt controls (Fig. 6L). *nrxn2a*^*tmr*^*2b*^*tmr*^ mutants still differed from wt controls (ART ANOVA, genotype x zone interaction: *F*(2,12) = 18.253, *p* = 0.0002), but showed clear improvement with bottom-dwelling reduced from 99 ± 0.3% in open field to 68 ± 5.5% when transferred to closed field.

In contrast, while transition to a closed/covered environment did not impact wt or *nrxn3a3b* mutants’ behaviour (Fig. 6O-R) and rescued/reduced *nrxn2a2b* defects, it did trigger significant alteration of all *nrxn1a1b* mutants’ behaviour (Fig. 6C-J). Comparison of the vertical distribution between wt and each *nrxn1a1b* line revealed a marked shift in shoal positioning (ART ANOVA, genotype x zone, *nrxn1a*^*full*^*1b*^*full*^, (*F*(2,12) = 22.551, *p* < 0.0001); *nrxn1a*^*part*^*1b*^*part*^*, F*(2,12) = 65.323, *p* < 0.0001; *nrxn1a*^*tmr*^*1b*^*tmr*^, *F*(2,12) = 8.095, *p* = 0.0059). Specifically, while these mutants behave within wt control ranges in the open field set-up, transfer to closed-field conditions triggered a significant surface-dwelling behaviour and reduction of exploration, with an average of 79 ± 4.6%, 88 ± 3% and 60 ± 6% of the shoal of *nrxn1a*^*full*^*b*^*full*^, *nrxn1a*^*par*^*1b*^*part*^ and *nrxn1a*^*tmr*^*1b*^*tmr*^ mutants distributed at the surface of the tanks during the assay (post-hoc pairwise comparison *vs*. wt: *p* = 0.0006, *p* < 0.0001 and *p* = 0.023, respectively). To our knowledge, this constitutes an undescribed behavioural response in such a paradigm, suggestive of profound and complex circuitry alterations, and may be particularly relevant given the strong association of human *NRXN1* mutations with schizophrenia, a condition characterised by altered sensorimotor gating and abnormal processing of environmental context.

In short, open to closed field transition did not affect wt or *nrxn3a3b* mutants’ behaviour but significantly rescued or reduced *nrxn2a2b*-induced anxiety-like behaviour, while, oppositely, significantly aggravated or triggered *nrxn1a1b* altered surface-dwelling behaviour.

### *nrxn3* does not trigger obvious abnormal change from early to adult stage

By comparison, *nrxn3a3b* double mutants developed normally and did not display overt alterations in exploratory, anxiety-like, or affiliative social behaviour (Figs. 4–6). Across novel tank diving, mirror-bitting, social interaction and open/closed shoal field tests, all *nrxn3a3b* mutants consistently performed within control ranges. These results indicate that *NRXN3* is either dispensable for the measured adult behaviours or functionally compensated in the studied mutants.

## Discussion

In this study, we generated the first complete collection of zebrafish neurexin knockout lines and the first systematic comparative phenotypic analysis, which underscores the distinct and non-redundant roles of neurexin paralogs in shaping adult behaviours. Interestingly, the absence of early obvious developmental defects, combined with the emergence of pronounced adult behavioural alterations, resembles the clinical trajectory of behavioural domains associated with many neurodevelopmental and psychiatric disorders such as schizophrenia, autism, and bipolar disorder, which often lack overt neuroanatomical hallmarks yet manifest with robust and rapidly progressing behavioural dysfunctions [1, 34]. These findings highlight both the conserved and complex role of neurexins in brain function and showcase zebrafish as an informative complementary model system to dissect the genetic and circuit-level mechanisms underlying behavioural domains associated with psychiatric conditions. The extreme and robust phenotypes observed with *nrxn1* and *nrxn2* LOF lines clearly exemplify the potential of the zebrafish for studying the role of genetics in mental health and behavioural disorders.

Our data show that *nrxn1* and *nrxn2*, but not *nrxn3*, are critical for the regulation of affiliative social behaviour, anxiety, and aggression in zebrafish. Loss of *nrxn2* produced a striking and penetrant anxiety-like phenotype, characterised by marked/persistent bottom-dwelling, reduced exploration, and frequent freezing episodes. Importantly, the freezing events observed were not limited to classic stress-induced immobility. Pattern analysis of the recordings revealed episodes of immobility followed by abrupt bursts of erratic hyperactivity. Furthermore, *nrxn2* mutants displayed marked hypersensitivity to PTZ challenges. Together, these findings suggest altered network excitability rather than purely heightened anxiety-like behaviour. Supporting our findings, Koh et al. while primarily investigating neuromuscular junction function, also reported that genetic disruption in exon 2 of *nrxn2a* induces anxiety- and freezing-like behaviours in zebrafish. Interestingly, these phenotypes attenuated across generations, whereas the behavioural phenotypes associated with our double mutant remain stable, which may reflect transcriptional adaptation or other compensatory mechanisms [11].

This interpretation is consistent with a role for *nrxn2* in synaptic transmission regulation and excitatory/inhibitory (E/I) balance. In mammals, *Nrxn2*/*NRXN2* is enriched in forebrain circuits involved in stress responsivity, and conditional deletion in rodents disrupts hippocampal circuitry and increases seizure susceptibility [35]. These observations are further supported by a substantial body of evidence linking *NRXN2* variants with E/I imbalance, epilepsy and neurodevelopmental disorders in rodents and patients [36–39]. Recent studies have even described *NRXN2* as an “inhibitory neurexin”, with its disruption significantly enhancing excitatory synaptic strength and release probability [40].

Importantly, neurexins, and notably *NRXN2*, are strongly associated with autism spectrum disorder and related psychiatric conditions in patients. Autism frequently presents with significant neuropsychiatric comorbidities, including elevated rates of anxiety (reported in ~42–56% of individuals), depression (12–70%), and epilepsy (8–30%) [37]. The combination of persistent anxiety-like behaviour and intermittent seizure-like episodes observed in *nrxn2* mutant zebrafish therefore aligns with behavioural domains that are commonly altered in human neuropsychiatric conditions. Altogether, these data reinforce the importance and translational relevance of *NRXN2*-dependent circuit regulation and identify behavioural dimensions particularly sensitive to its loss.

Although direct confirmation and characterisation of seizure activity in zebrafish will require electrophysiology or calcium imaging as well as detailed spatiotemporal expression mapping, the convergence of behavioural, pharmacological, and cross-species evidence supports a conserved role for *NRXN2* in maintaining inhibitory circuit stability. While our data should not be interpreted as modelling a specific clinical entity such as autism, they identify behavioural domains sensitive to *NRXN2* alterations and provide a mechanistically grounded platform for future circuit-level and pharmacological investigations.

Conversely, *nrxn1* deletion did not elicit strong anxiety-like behaviour in zebrafish but instead induced profound alterations in affiliative social behaviour, aggression, and an unusual surface-dwelling phenotype that was exacerbated under closed-field conditions. These results resonate with the fact that *NRXN1* has been strongly associated with schizophrenia, which also develops with significant social interaction alterations and aggression modulations in patients [1, 4, 41]. Interestingly, similar social behavioural alterations have been identified in mouse *neurexin-1* mutant lines [42, 43].

It is noteworthy that these phenotype divergences between *nrxn1* and *nrxn2* mutants are of particular interest. Confinement alleviated anxiety-like behaviours in *nrxn2* mutants but paradoxically triggered aberrant surface-dwelling in *nrxn1* mutants. This suggests that different neurexin paralogs gate how environmental cues are integrated into behavioural output. In humans, *NRXN1* deletions have been strongly linked with schizophrenia and abnormal sensory gating, while *NRXN2* has been linked with heightened anxiety and ASD-like features. Our zebrafish data therefore support the notion that *nrxn1* and *nrxn2* act through distinct neural pathways that differentially process environmental and contextual information, which could explain why different paralogs are associated with different psychiatric conditions.

Another striking observation is that none of the zebrafish *nrxn* mutants displayed gross developmental or morphological defects during embryonic or larval stages. Brain volume, gross anatomy, and basic locomotor responses were all indistinguishable from wild-type controls. Yet, profound phenotypes emerged at later developmental stages. This mirrors the clinical paradox of psychiatric disorders, which are widely recognised to have early neurodevelopmental origins yet lack obvious developmental abnormalities. This “silent developmental trajectory” supports the hypothesis that neurexin dysfunction perturbs synaptic wiring or plasticity in ways that remain latent until circuits mature or environmental demands and social interaction/needs increase. Zebrafish, with their optical accessibility and powerful in vivo imaging tools early in life, are ideally suited to investigate the precise timing and mechanisms of such latent circuit disruptions.

By contrast, *nrxn3* mutants did not display obvious abnormalities in the behavioural assays performed. However, this does not exclude a possible effect on behavioural domains not captured by the paradigms used here, and the absence of detectable phenotypes may reflect partial functional redundancy via other neurexin paralogues. Indeed, *nrxn3* loss may be compensated by the presence of *NRXN1* or *NRXN2* within shared neural circuits.

Detailed spatial and temporal expression maps of zebrafish neurexin paralogues across development and adulthood have not yet been systematically characterised, which limits the ability to directly test these hypotheses. More broadly, comprehensive spatiotemporal mapping of neurexin paralogues and their isoforms in the zebrafish nervous system will be important to better interpret the paralog-specific behavioural divergence observed here and to refine the mechanistic understanding of neurexin-dependent behavioural regulation.

## Limitations

A limitation of the present study regarding its relevance to human diseases is that behavioural analyses were performed exclusively in homozygous mutants, whereas most pathogenic *NRXN* variants in humans act as heterozygous risk factors within complex genetic architectures. The presented models should therefore be interpreted as defining behavioural consequences of complete paralog-specific neurexin loss, rather than as direct genetic replicas of human heterozygous conditions. We deliberately adopted a full loss-of-function approach to robustly delineate paralog-dependent behavioural domains and establish a clear functional framework. Future studies investigating heterozygous states, isoform-specific contributions, and pharmacological interactions will be essential to refine the translational relevance of these findings.An additional limitation relates to the interpretation of the phenotypic differences observed between the full genomic deletions (*full*) and the transmembrane-domain (*tmr*) mutants. While the full genomic deletions are expected to eliminate gene function entirely, deletion of the transmembrane domain may still allow production of a near full-length protein, lacking membrane anchoring and thereby likely to be secreted in the associated synaptic clefts. Such a soluble extracellular neurexin fragment could interfere with synaptic interactions and circuit functions, thereby contributing to the stronger behavioural phenotypes observed in some *tmr* mutants. Although this hypothesis remains speculative and will require direct biochemical validation, the broadly similar phenotypes observed across both mutation strategies strongly support the specificity of the neurexin-dependent effects reported here. Future work will be required to determine whether truncated extracellular neurexin fragments are produced and to clarify their potential impact on synaptic organisation and circuit function.

Finally, the absence of obvious embryonic or larval phenotypes should not be taken to mean that early neural development is entirely unaffected by neurexin loss. Our analyses were designed to detect gross morphological, locomotor, and broad behavioural abnormalities, but would not capture subtle synaptic, circuit-level, or cell-type-specific defects. It is therefore plausible that neurexin deficiency perturbs early network assembly, synaptic maturation, or activity-dependent refinement in ways that remain silent until later developmental stages, when behavioural demands increase. Future studies using whole-brain activity mapping, calcium imaging, electrophysiology, and higher-resolution/speed motor analyses will be required to define the timing and nature of these possible latent alterations.

## Conclusion

In summary, our study introduces the first complete set of zebrafish mutants spanning the entire *neurexin* gene family. Despite showing no overt neurodevelopmental or motor defects during embryonic and larval stages, these lines reveal pronounced and paralog-specific behavioural disturbances that emerge only in later stages of life. *nrxn1* loss in zebrafish primarily alters affiliative social behaviour and aggression, while *nrxn2* triggers severe anxiety-like behaviours and seizure-like events. *nrxn3* loss, however, appears dispensable in all domains tested, which is consistent with a less robust association between *NRXN3* and mental health disorders in humans.

These phenotype trajectories in zebrafish mirror a central challenge in human mental health research: most psychiatric and neurodevelopmental disorders are widely accepted to originate early in life, yet often present without clear, detectable abnormalities during early development. By uncovering dramatic adult phenotypes in the absence of obvious early defects, these mutants create a unique opportunity to explore the subtle, progressive, and circuit-level mechanisms that unfold between early development and behavioural manifestation. The optical accessibility, genetic tractability, and whole-brain imaging capacity of the zebrafish make it an exceptionally powerful model to dissect how early molecular perturbations in synaptic adhesion genes translate into late-onset behavioural pathology. As such, these *nrxn* mutants open a new avenue to investigate the developmental timing, network rewiring, and mechanistic origins of mental disorders, with direct relevance for understanding disease onset and for identifying novel therapeutic windows.

## Supplementary information

Supplemental video S1

Supplemental video S2

Supplemental video S3

Supplemental video S4

Supplemental video S5

SUPPLEMENTAL MATERIAL

Supplemental File S1

Supplemental File S2

Supplemental File S3
