# Supplementary material for: Towards modelling mental disorders in zebrafish. Neurexins severely modulate anxiety, affiliative social behaviour and aggression
Source: Mol Psychiatry. 2026 Jun 26;31(11):6517–31. doi: 10.1038/s41380-026-03721-1 (PMC13668736; doi:10.1038/s41380-026-03721-1)
Supplement: Supplementary file 1 [file 41380_2026_3721_MOESM6_ESM.pdf]

Towards modelling mental disorders in zebrafish. Neurexins severely modulate anxiety, social behaviours and aggression.

Nguyen Q[1*], Guo F.[1*], Das J.[1], Hatzimanolis O. [1], Mowry B.[3], Cristino A. [1], Giacomotto J.[1,2,3,4#]

Table of contents:

**Supplemental Table S1.**	Page 2
**Supplemental Table S2.**	Page 3
**Supplemental Table S3.**	Page 4
**Supplemental Figure S1.**	Page 5
**Supplemental Figure S2.**	Page 6
**Supplemental figure S3.**	Page 7
**Supplemental figure S4.**	Page 8
**Supplemental Video S1.**	Page 9
**Supplemental Video S2.**	Page 9
**Supplemental Video S3.**	Page 9
**Supplemental Video S4.**	Page 9
**Supplemental Video S5.**	Page 9
**Supplemental Files S1.**	Page 9
**Supplemental Files S2.**	Page 9
**Supplemental Files S3.**	Page 9

Supplemental Table S1. CRISPR/Cas9 guide RNAs and oligonucleotides used in this study.

GENE	Oligo	Oligo Name	Description	Ordered sgRNA/CRISPR Oligo	Genomic Target Sequence	Protocol used (Tromp et al. 2021)
nrxn1a	C1	41_g1a-1	Promoter region	taatacgactcactataGGTGGTGCAGCATTCAAACAGTTTTAGAGCTAGAAATAGCAAG	GGTGGTGCAGCATTCAAACA	Extension/Annealing
	C2	42_g1a-2	Promoter region	taatacgactcactataGGAAACAGAGAAGACCATACGTTTTAGAGCTAGAAATAGCAAG	GGAAACAGAGAAGACCATAC	Extension/Annealing
	C3	45_g1a-5	3'UTR region	TAATACGACTCACTATAGGGCCATTCGGAATAGATTGAGGGTTTTAGAGCTAGAAATAGCAAG	GGGCCATTCGGAATAGATTG	Extension/Annealing
	C4	46_g1a-6	3'UTR region	TAATACGACTCACTATAGGCAGTATTGCGCGGATGTTTGGGTTTTAGAGCTAGAAATAGCAAG	GGCAGTATTGCGCGGATGTT	Extension/Annealing
	C5	43_g1a-3	Exon 7	taatacgactcactataGGCCTCTTGCGTATATCCAGGTTTTAGAGCTAGAAATAGCAAG	GGCCTCTTGCGTATATCCAG	Extension/Annealing
	C6	44_g1a-4	Exon 7	taatacgactcactataGGCAGGTGATCCAGGAAGATGTTTTAGAGCTAGAAATAGCAAG	GGCAGGTGATCCAGGAAGAT	Extension/Annealing
	C7	85_nrxn1a-TransMB_CRISPR_01	5' of TransMembrane domain sequence	taatacgactcactataGGTCGGGTAACCCTTGGCCTgttttagagctagaaatagcaag	GCTCGGGTAACCCTTGGCCT	Extension/Annealing
	C8	86_nrxn1a-TransMB_CRISPR_02	5' of TransMembrane domain sequence	taatacgactcactataGGACTCCCGAATCACCTCTgttttagagctagaaatagcaag	GGACTCCCGAATCACCTCT	Extension/Annealing
	C9	87_nrxn1a-TransMB_CRISPR_03	3' of TransMembrane domain sequence	taatacgactcactataGGACGAAGGGTCTTACCACGgttttagagctagaaatagcaag	CGACGAAGGGTCTTACCACG	Extension/Annealing
	C10	88_nrxn1a-TransMB_CRISPR_04	3' of TransMembrane domain sequence	taatacgactcactataGGCAGCTCCATTGGGCTGCGgttttagagctagaaatagcaag	GGCAGCTCCATTGGGCTGCG	Extension/Annealing
nrxn1b	C11	47_g1b-1	Promoter region	taatacgactcactataGGCCAAGGTGCTGCAAAGCGGTTTTAGAGCTAGAAATAGCAAG	GTCCAAGGTGCTGCAAAGCG	Extension/Annealing
	C12	48_g1b-2	Promoter region	taatacgactcactataGGTGATGTGAATCTGGCGGGGTTTTAGAGCTAGAAATAGCAAG	CGTGATGTGAATCTGGCGGG	Extension/Annealing
	C13	50_g1b-5	3'UTR region	TAATACGACTCACTATAGGCATAGTAATGGGGGGAAATGGGTTTTAGAGCTAGAAATAGCAAG	GGCATAGTAATGGGGGGAAA	Extension/Annealing
	C14	51_g1b-6	3'UTR region	taatacgactcactataAGTCAAGAGCGTGGGTGAGGGTTTTAGAGCTAGAAATAGCAAG	AGTCAAGAGCGTGGGTGAGG	Extension/Annealing
	C15	n1bgRNA1exon7	Exon 7	TAATACGACTCACTATAGGCGGCTCTTTACCGCAGGAGTTTTAGAGCTAGAAATAGCAAG	GGCGGCTCTTTACCGCAGGA	Extension/Annealing
	C16	n1bgRNA2exon7	Exon 7	TAATACGACTCACTATAGGAGCGCCCTGCTGAACTACGTTTTAGAGCTAGAAATAGCAAG	GGAGCGCCCTGCTGAACTAC	Extension/Annealing
	C17	89_nrxn1b-TransMB_CRISPR_01	5' of TransMembrane domain sequence	taatacgactcactataGGCTGATCGCGGGGCCTGTGgttttagagctagaaatagcaag	TGCTGATCGCGGGGCCTGTG	Extension/Annealing
	C18	90_nrxn1b-TransMB_CRISPR_02	5' of TransMembrane domain sequence	taatacgactcactataGGGAGTCCAACGGCACCACAgttttagagctagaaatagcaag	GGGAGTCCAACGGCACCACA	Extension/Annealing
	C19	91_nrxn1b-TransMB_CRISPR_03	3' of TransMembrane domain sequence	taatacgactcactataGGACGAGGGGTCGTACCATGgttttagagctagaaatagcaag	AGACGAGGGGTCGTACCATG	Extension/Annealing
	C20	92_nrxn1b-TransMB_CRISPR_04	3' of TransMembrane domain sequence	taatacgactcactataGGTTTCTGCTCTCGTCCACAgttttagagctagaaatagcaag	AGTTTCTGCTCTCGTCCACA	Extension/Annealing
nrxn2a	C21	c70_nrxn2A_CRISPR-01_TOP	5'UTR region	TAATACGACTCACTATAGGAGTGCACCTGCATTCCACGTTTTAGAGCTAGAAATAGCAAGTTAAAATAAGGCTAGTCCGTTATCAACTTGAAAAAGTGGCACCGAGTCGGTGCTTTT	GAAGTGCACCTGCATTCCAC	Annealing
	C22	c71_nrxn2A_CRISPR-01_BOTTOM	5'UTR region	AAAAGCACCGACTCGGTGCCACTTTTTCAAGTTGATAACGGACTAGCCTTATTTTAACTTGCTATTTCTAGCTCTAAAACGTGGAATGCAGGTGCACTCCTATAGTGAGTCGTATTA	ottom oligos for direct annealin	Annealing
	C23	c72_nrxn2A_CRISPR-02_TOP	5'UTR region	TAATACGACTCACTATAGGTAATCCTACTTTCACCAGGTTTTAGAGCTAGAAATAGCAAGTTAAAATAAGGCTAGTCCGTTATCAACTTGAAAAAGTGGCACCGAGTCGGTGCTTTT	AGTAATCCTACTTTCACCAG	Annealing
	C24	c73_nrxn2A_CRISPR-02_BOTTOM	5'UTR region	AAAAGCACCGACTCGGTGCCACTTTTTCAAGTTGATAACGGACTAGCCTTATTTTAACTTGCTATTTCTAGCTCTAAAACCTGGTGAAAGTAGGATTACCTATAGTGAGTCGTATTA	ottom oligos for direct annealin	Annealing
	C25	c27_nrxn2a CRISPR-01	5' of TransMembrane domain sequence	TAATACGACTCACTATAGGTAGGTTAGACCTGGAGTGGTTTTAGAGCTAGAAATAGCAAG	GGTAGGTTAGACCTGGAGTG	Extension/Annealing
	C26	c28_nrxn2a CRISPR-02	5' of TransMembrane domain sequence	TAATACGACTCACTATAGGGCCGTCCAGGTGCCGTGGGTTTTAGAGCTAGAAATAGCAAG	GGGCCGTCCAGGTGCCGTGG	Extension/Annealing
	C27	c29_nrxn2a CRISPR-03	3' of TransMembrane domain sequence	TAATACGACTCACTATAGGTGGTGGTTTTAGCCACGGGTTTTAGAGCTAGAAATAGCAAG	GGTGGTGGTTTTAGCCACGG	Extension/Annealing
	C28	c30_nrxn2a CRISPR-04	3' of TransMembrane domain sequence	TAATACGACTCACTATAGGCACCGTTGCTTTCAGAGCGTTTTAGAGCTAGAAATAGCAAG	GGCACCGTTGCTTTCAGAGC	Extension/Annealing
nrxn2b	C29	c74_nrxn2b_CRISPR-01_TOP	5'UTR region	TAATACGACTCACTATAGGGTGTTCGCTGCTAGTCTCGTTTTAGAGCTAGAAATAGCAAGTTAAAATAAGGCTAGTCCGTTATCAACTTGAAAAAGTGGCACCGAGTCGGTGCTTTT	CGGTGTTCGCTGCTAGTCTC	Annealing
	C30	c75_nrxn2b_CRISPR-01_BOTTOM	5'UTR region	AAAAGCACCGACTCGGTGCCACTTTTTCAAGTTGATAACGGACTAGCCTTATTTTAACTTGCTATTTCTAGCTCTAAAACGAGACTAGCAGCGAACACCCTATAGTGAGTCGTATTA	ottom oligos for direct annealin	Annealing
	C31	c76_nrxn2b_CRISPR-02_TOP	5'UTR region	TAATACGACTCACTATAGGGGCAGGCAGTGTTGACGGGTTTTAGAGCTAGAAATAGCAAGTTAAAATAAGGCTAGTCCGTTATCAACTTGAAAAAGTGGCACCGAGTCGGTGCTTTT	GGGGCAGGCAGTGTTGACGG	Annealing
	C32	c77_nrxn2b_CRISPR-02_BOTTOM	5'UTR region	AAAAGCACCGACTCGGTGCCACTTTTTCAAGTTGATAACGGACTAGCCTTATTTTAACTTGCTATTTCTAGCTCTAAAACCCGTCAACACTGCCTGCCCCTATAGTGAGTCGTATTA	ottom oligos for direct annealin	Annealing
	C33	c31_nrxn2b CRISPR-01	5' of TransMembrane domain sequence	TAATACGACTCACTATAGGGCGGCATCGACACATCCCGTTTTAGAGCTAGAAATAGCAAG	GGGCGGCATCGACACATCCC	Extension/Annealing
	C34	c32_nrxn2b CRISPR-02	5' of TransMembrane domain sequence	TAATACGACTCACTATAGGTCTGTAGTGGGCACATGGGTTTTAGAGCTAGAAATAGCAAG	GGTCTGTAGTGGGCACATGG	Extension/Annealing
	C35	c33_nrxn2b CRISPR-03	3' of TransMembrane domain sequence	TAATACGACTCACTATAGGTGCCCGTCTTGGCAGCGCGTTTTAGAGCTAGAAATAGCAAG	GGTGCCCGTCTTGGCAGCGC	Extension/Annealing
	C36	c34_nrxn2b CRISPR-04	3' of TransMembrane domain sequence	TAATACGACTCACTATAGGGCTGTTTCTCCTTCACTAGTTTTAGAGCTAGAAATAGCAAG	GGGCTGTTTCTCCTTCACTA	Extension/Annealing
nrxn3a	C37	C92-Nrxn3a-Crispr03-Top	5'UTR region	TAATACGACTCACTATAGGGCTAGTAGCATACTAAGGGTTTTAGAGCTAGAAATAGCAAGTTAAAATAAGGCTAGTCCGTTATCAACTTGAAAAAGTGGCACCGAGTCGGTGCTTTT	GTGCTAGTAGCATACTAAGGAGG	Annealing
	C38	C93-Nrxn3a-Crispr03-Bottom	5'UTR region	AAAAGCACCGACTCGGTGCCACTTTTTCAAGTTGATAACGGACTAGCCTTATTTTAACTTGCTATTTCTAGCTCTAAAACCCTTAGTATGCTACTAGCCCTATAGTGAGTCGTATTA	ottom oligos for direct annealin	Annealing
	C39	C94-Nrxn3a-Crispr04-Top	5'UTR region	TAATACGACTCACTATAGGCTGTGTTGGCAGTGACATGTTTTAGAGCTAGAAATAGCAAGTTAAAATAAGGCTAGTCCGTTATCAACTTGAAAAAGTGGCACCGAGTCGGTGCTTTT	GCCTGTGTTGGCAGTGACATAGG	Annealing
	C40	C95-Nrxn3a-Crispr04-Bottom	5'UTR region	AAAAGCACCGACTCGGTGCCACTTTTTCAAGTTGATAACGGACTAGCCTTATTTTAACTTGCTATTTCTAGCTCTAAAACATGTCACTGCCAACACAGCCTATAGTGAGTCGTATTA	ottom oligos for direct annealin	Annealing
	C41	c40_nrxn3a CRISPR-03	3' of TransMembrane domain sequence	TAATACGACTCACTATAGGGCACTGCGCCAAGAGAGAGTTTTAGAGCTAGAAATAGCAAG	GGGCACTGCGCCAAGAGAGAGGG	Extension/Annealing
	C42	C46-nrxn3a-CRISPR-01-TOP	5' of TransMembrane domain sequence	TAATACGACTCACTATAGGGTACATTACGGAACATGGGTTTTAGAGCTAGAAATAGCAAGTTAAAATAAGGCTAGTCCGTTATCAACTTGAAAAAGTGGCACCGAGTCGGTGCTTTT	GGGTACATTACGGAACATGG	Annealing
	C43	C47-nrxn3a-CRISPR-01-Bottom	5' of TransMembrane domain sequence	AAAAGCACCGACTCGGTGCCACTTTTTCAAGTTGATAACGGACTAGCCTTATTTTAACTTGCTATTTCTAGCTCTAAAACCCATGTTCCGTAATGTACCCTATAGTGAGTCGTATTA	ottom oligos for direct annealin	Annealing
	C44	c64_nrxn3a CRISPR-05_TOP	5' of TransMembrane domain sequence	TAATACGACTCACTATAGGGCATCAGGCGGGTTCCGGGTTTTAGAGCTAGAAATAGCAAGTTAAAATAAGGCTAGTCCGTTATCAACTTGAAAAAGTGGCACCGAGTCGGTGCTTTT	GGGCATCAGGCGGGTTCCGG	Annealing
	C45	c65_nrxn3a CRISPR-05_BOTTOM	5' of TransMembrane domain sequence	AAAAGCACCGACTCGGTGCCACTTTTTCAAGTTGATAACGGACTAGCCTTATTTTAACTTGCTATTTCTAGCTCTAAAACCCGGAACCCGCCTGATGCCCTATAGTGAGTCGTATTA	ottom oligos for direct annealin	Annealing
	C46	c66_nrxn3a CRISPR-06_TOP	3' of TransMembrane domain sequence	TAATACGACTCACTATAGGACGAGGGGTCCTACCAAGGTTTTAGAGCTAGAAATAGCAAGTTAAAATAAGGCTAGTCCGTTATCAACTTGAAAAAGTGGCACCGAGTCGGTGCTTTT	GGACGAGGGGTCCTACCAAG	Annealing
	C47	c67_nrxn3a CRISPR-06_BOTTOM	3' of TransMembrane domain sequence	AAAAGCACCGACTCGGTGCCACTTTTTCAAGTTGATAACGGACTAGCCTTATTTTAACTTGCTATTTCTAGCTCTAAAACCTTGGTAGGACCCCTCGTCCTATAGTGAGTCGTATTA	ottom oligos for direct annealin	Annealing
	C48	c68_nrxn3a CRISPR-07_TOP	3' of TransMembrane domain sequence in CDS	TAATACGACTCACTATAGGCATTGCTCTGCGCCGAGTGTTTTAGAGCTAGAAATAGCAAGTTAAAATAAGGCTAGTCCGTTATCAACTTGAAAAAGTGGCACCGAGTCGGTGCTTTT	GCCATTGCTCTGCGCCGAGT	Annealing
	C49	c69_nrxn3a CRISPR-07_BOTTOM	3' of TransMembrane domain sequence in CDS	AAAAGCACCGACTCGGTGCCACTTTTTCAAGTTGATAACGGACTAGCCTTATTTTAACTTGCTATTTCTAGCTCTAAAACACTCGGCGCAGAGCAATGCCTATAGTGAGTCGTATTA	ottom oligos for direct annealin	Annealing
nxn3b	C50	C102-nrxn3b-CRISPR03-Top	5'UTR region	TAATACGACTCACTATAGGCGACAACAAGGGCAAAGAGTTTTAGAGCTAGAAATAGCAAGTTAAAATAAGGCTAGTCCGTTATCAACTTGAAAAAGTGGCACCGAGTCGGTGCTTTT	AGCGACAACAAGGGCAAAGAGGG	Annealing
	C51	C103-nrxn3b-CRISPR03-Bottom	5'UTR region	AAAAGCACCGACTCGGTGCCACTTTTTCAAGTTGATAACGGACTAGCCTTATTTTAACTTGCTATTTCTAGCTCTAAAACTCTTTGCCCTTGTTGTCGCCTATAGTGAGTCGTATTA	ottom oligos for direct annealin	Annealing
	C52	C104-nrxn3b-CRISPR04-Top	5'UTR region	TAATACGACTCACTATAGGGAACAGCTACGACTGGGGGTTTTAGAGCTAGAAATAGCAAGTTAAAATAAGGCTAGTCCGTTATCAACTTGAAAAAGTGGCACCGAGTCGGTGCTTTT	TGGAACAGCTACGACTGGGGAGG	Annealing
	C53	C105-nrxn3b-CRISPR04-Bottom	5'UTR region	AAAAGCACCGACTCGGTGCCACTTTTTCAAGTTGATAACGGACTAGCCTTATTTTAACTTGCTATTTCTAGCTCTAAAACCCCCAGTCGTAGCTGTTCCCTATAGTGAGTCGTATTA	ottom oligos for direct annealin	Annealing
	C54	c42_nrxn3b CRISPR-01	5' of TransMembrane domain sequence	TAATACGACTCACTATAGGGGGCACTCTCCTGATACCGTTTTAGAGCTAGAAATAGCAAG	GGGGGCACTCTCCTGATACC	Extension/Annealing
	C55	c43_nrxn3b CRISPR-02	5' of TransMembrane domain sequence	taatacgactcactataGGTGTGATGTACGGCCCCTTgttttagagctagaaATAGCAAG	TGTGTGATGTACGGCCCCTT	Extension/Annealing
	C56	c44_nrxn3b CRISPR-03	3' of TransMembrane domain sequence	taatacgactcactataGGAATTACTGATGTAGTTCCgttttagagctagaaATAGCAAG	CGAATTACTGATGTAGTTCC	Extension/Annealing
	C57	c45_nrxn3b CRISPR-04	3' of TransMembrane domain sequence	taatacgactcactataGGCTTTGGGTCTCTTGTTACgttttagagctagaaATAGCAAG	GTCTTTGGGTCTCTTGTTAC	Extension/Annealing

Supplemental Table S2. NRXN genotyping primers used in this study.

	Gene name	Primer name	Database name	Sequence (5'– 3')	Direction	Description / Position	Mutant Alelle Amplicon (bp)	WT Alelle Amplicon (bp)	Primers Pair
nrxn1	*nrxn1a*^{full}	P1	214-nrxn1aFULLdelF1	GCATAGACTTCGAGTGTCATG	Forward	nrxn1a-5'UTR region	307	0	P1xP2
		P2	215-nrxn1aFULLdelR1	CACAGATAGTCTTTGCACCG	Reverse	nrxn1a-3'UTR region			
		P3	179-nrxn1a-DEL-FORW	TAAACGCTCTCTCTTTCTCC	Forward	nrxn1a-5' transmembrane domain	0	327	P3xP4
		P4	180-nrxn1a-DEL-Rev	GTACTCCTTGTCCTTGTTCT	Reverse	nrxn1a-3' transmembrane domain			
	nrxn1a^{part}	P9	226-nrxn1A_PartialF5	CTTGACTTACAGTGTTCAGCC	Forward	nrxn1a-5'UTR region	445	0	P9xP10
		P10	228-nrxn1A_PartialR5	TTTCCGAGGAGCAATTGTTC	Reverse	nrxn1a-Intron 8			
		P11	151-nrxn1aGENO_For	CCTTACGAGGGGGACCAAAG	Forward	nrxn1a-Intron 7	0	278	P11xP10
	nrxn1a^{tmr}	P3	179-nrxn1a-DEL-FORW	TAAACGCTCTCTCTTTCTCC	Forward	nrxn1a-5' transmembrane domain	203	327	P3xP4
		P4	180-nrxn1a-DEL-Rev	GTACTCCTTGTCCTTGTTCT	Reverse	nrxn1a-3' transmembrane domain			
	nrxn1b^{full}	P5	213-nrxn1bFULLdelF1	GCTTAGTTTTGCTGTCATGTGTAC	Forward	nrxn1b-5'UTR region	264	0	P5xP6
		P6	217-nrxn1bPARTIALdelR1	CGTAAGTTATACAAGAAACAGCGAG	Reverse	nrxn1b-3'UTR region			
		P7	181-nrxn1b-DEL-FORW	GACCCTGTGTTGTTTTGTTC	Forward	nrxn1Bb-5' transMembrane domain	0	278	P7xP8
		P8	182-nrxn1b-DEL-Rev	CGCTGTCGTCGTCTGCAGTG	Reverse	nrxn1Bb-3' transMembrane domain			
	nrxn1b^{part}	P5	213-nrxn1bFULLdelF1	GCTTAGTTTTGCTGTCATGTGTAC	Forward	nrxn1b-5'UTR region	390	0	P12xP5
		P12	161-Rev_Nrxn1b_ES	CTGCCATCGAAAGAGAGGAT	Reverse	nrxn1b-exon 8			
		P13	162-For_Nrxn1b_GENO	CAGTGTGGATGTCCGGCG	Forward	nrxn1b-exon 7	0	281	P12xP13
	nrxn1b^{tmr}	P7	181-nrxn1b-DEL-FORW	GACCCTGTGTTGTTTTGTTC	Forward	nrxn1b-5' transMembrane domain	286	278 (MslI digestion - 182 + 96)	P7xP8 (Unique MslI site on wt)
		P8	182-nrxn1b-DEL-Rev	CGCTGTCGTCGTCTGCAGTG	Reverse	nrxn1b-3' transMembrane domain			
nrxn2	*nrxn2a*^{full}	P14	219-nrxn2A-Full_F	GCTACATGTGTGAACATGCC	Forward	nrxn2a-5'UTR region	325	0	P14xP15
		P15	210-nrxn2aDelR2	CATCAGTACCTTCTCCTCTGTC	Reverse	nrxn2a-3' transMembrane domain			
		P16	245-nrxn2aDelF3	CCTTCAGACCACCAAAGAGTCC	Forward	nrxn2a-exon 24	0	711	P15xP16
	nrxn2a^{tmr}	P21	209-nrxn2aDelF2	CCTGGTGTAGGCAAAAGTGG	Forward	nrxn2a-5' transMembrane domain	219	524	P21xP15
		P15	210-nrxn2aDelR2	CATCAGTACCTTCTCCTCTGTC	Reverse	nrxn2a-3' transMembrane domain			
		P16	245-nrxn2aDelF3	CCTTCAGACCACCAAAGAGTCC	Forward	nrxn2a-exon 24	403	711	P15xP16
	nrxn2b^{full}	P17	233-nrxn2bFULLdelF2	TTCCTGCGTGATTTATTCTCCC	Forward	nrxn2b-exon1	345	0	P17xP18
		P18	288-nrxn2bDEL_R	ACTCTCGCACTCTTTAGCTC	Reverse	nrxn2b-exon7			
		P19	287-nrxn2bDEL_F	GCAGAGTCTGCTTCATTAGC	Forward	nrxn2b-exon1	0	446	P19xP10
		P20	234-nrxn2bFULLdelR1	CCAAAACAGCTACCTACATCTAACG	Reverse	nrxn2b-exon1			
	nrxn2b^{tmr}	P22	211-nrxn2bDelF2	GCTCAAACCGGGCATCAATG	Forward	nrxn2b-5' transMembrane domain	284	527	P22xP23
		P23	212-nrxn2bDelR2	CCAGTCCTCATCCTATCTCTCTC	Reverse	nrxn2b-3' transMembrane domain			
		P24	250-nrxn2bDelF3	CATCCTCATCCTGCTGTACG	Forward	nrxn2b-exon 7	0	251	P23xP24
nrxn3	*nrxn3a*^{full}	P25	235-nrxn3AFulldelF2	GGTGGAACAATACAAGAGGC	Forward	nrxn3a-5'UTR region	232	0	P25xP26
		P26	206-nrxn3aDelR1	CCTTTCGCGGTATGCTTACT	Reverse	nrxn3a-3' transMembrane domain			
		P27	205-nrxn3aDelF1	CGACATAGTCTTGCTTCCAT	Forward	nrxn3a-5' transMembrane domain	0	570	P26xP27
	nrxn3a^{tmr}	P27	205-nrxn3aDelF1	CGACATAGTCTTGCTTCCAT	Forward	nrxn3a-3' transMembrane domain	380	570	P26xP27
		P26	206-nrxn3aDelR1	CCTTTCGCGGTATGCTTACT	Reverse	nrxn3a-5' transMembrane domain			
	nrxn3b^{full}	P28	264-nrxn3bFULLdelF4	GAGAGATTAACTATTCAGGGCAGG	Forward	nrxn3b-5'UTR region	208	0	P28xP29
		P29	208-nrxn3bDelR1	CTCAGATGTTGCTCTGTCTC	Reverse	nrxn3b-3' transMembrane domain			
		P30	207-nrxn3bDelF1	CCAAAATCCCAGCAGGGAAA	Forward	nrxn3b-5' transMembrane domain	0	503	P29xP30
	nrxn3b^{tmr}	P30	207-nrxn3bDelF1	CCAAAATCCCAGCAGGGAAA	Forward	nrxn3b-5' transMembrane domain	188	503	P29xP30
		P29	208-nrxn3bDelR1	CTCAGATGTTGCTCTGTCTC	Reverse	nrxn3b-3' transMembrane domain			

Supplemental Table S3. Genomic region coordinates associated with analyses and schematics presented in Figure 1E and Figure 1F.

Name of region	chromosome	Start genomic coordinates	End genomic coordinates	strand
nrxn1a-alpha	12	24354519	24549571	+
nrxn1a-beta	12	24565477	24628947	+
nrxn1b-alpha	13	740461	763779	+
nrxn1b-beta	13	784968	804958	+
nrxn2a-alpha	21	28008862	28192946	-
nrxn2a-beta	21	27643860	27725919	-
nrxn2b-alpha	7	459338	506340	+
nrxn2b-beta	7	568818	605086	+
nrxn3a-alpha	17	16994865	17433482	-
nrxn3a-beta	17	16913503	16965809	-
nrxn3b-alpha	20	5581690	5700369	+
nrxn3b-beta	20	5729399	5965307	+

Supplemental Figure S1. Larval swimming patterns and locomotor activity of wild-type controls versus the different neurexin mutant lines presented in this study. Total distance moved (mm) of 6 dpf larvae (plotted per 5-second bin) over 32-minute recordings. No significant differences were observed between mutants or controls.

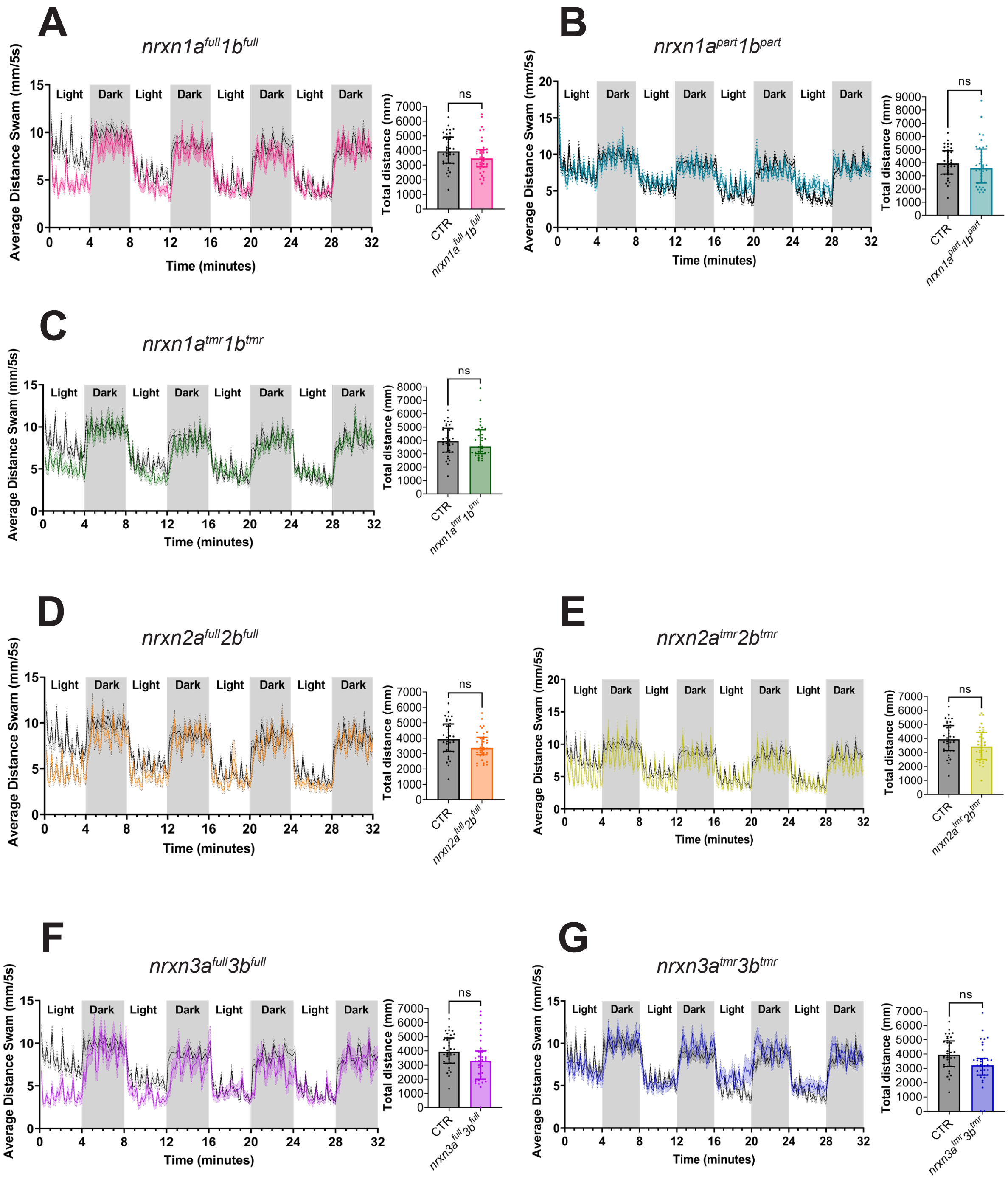

Supplemental Figure S2. Larval swimming patterns and locomotor activity of wild-type controls versus the different neurexin mutant lines presented in this study. Total distance moved (mm) of 7 dpf larvae (plotted per 5-second bin) over 32-minute recordings. No significant differences were observed between mutants or controls.

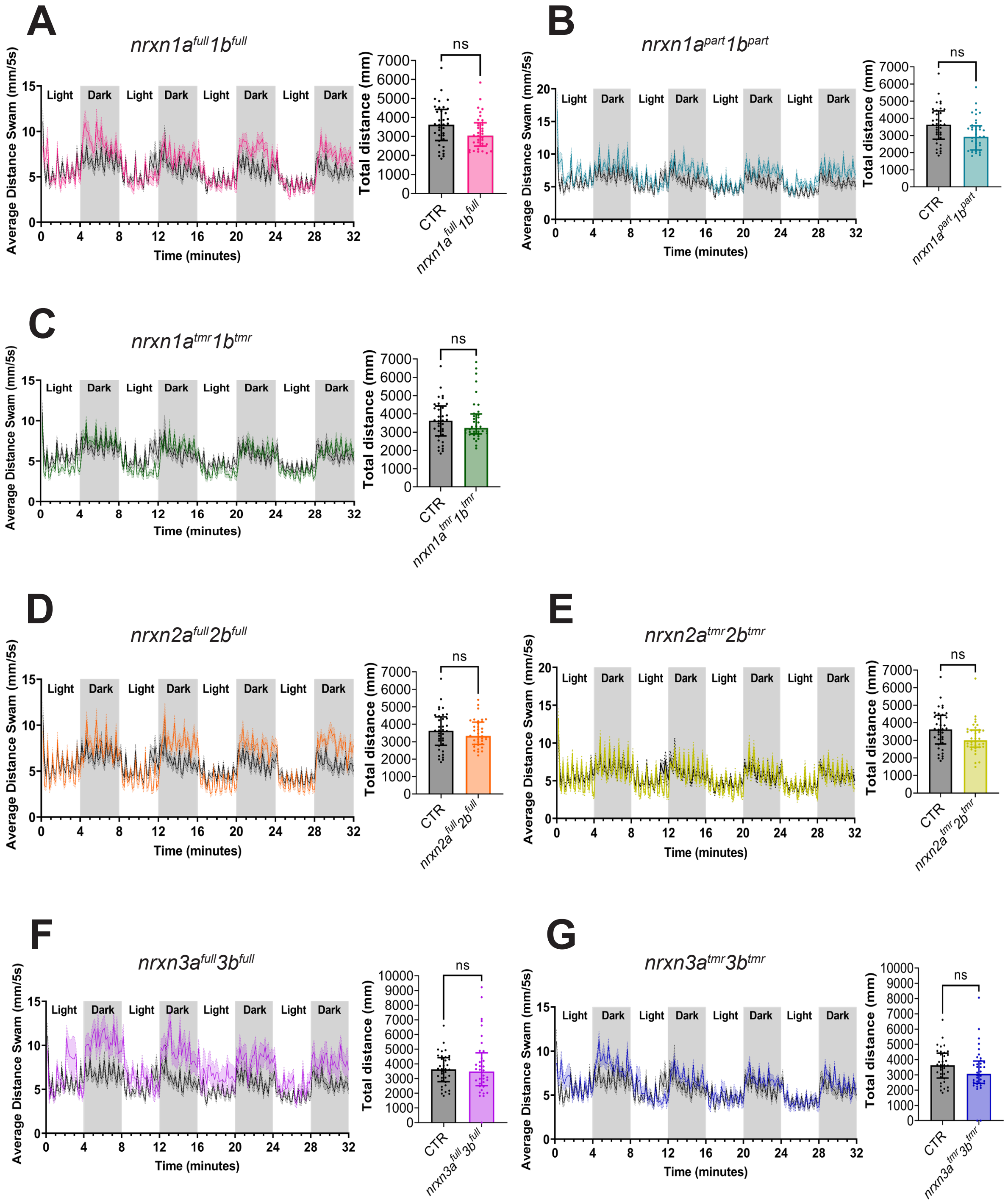

Supplemental figure S3. *nrxn2a^tmr^2b^tmr^* mutants exhibit some freezing episodes associated with bursts of erratic hyperactivity, and enhanced PTZ-induced seizure susceptibility. A, Percentage of *nrxn2a^{tmr}2b^{tmr}* fish exhibiting freezing behaviour in novel tank diving assays presented in figure 4. The inset highlights/quantify the percentage of animals displaying freezing events associated with hyperactive/erratic bursts relative to the total number of fish showing freezing behavior. **B,** Analysis of the distribution of the two different freezing episodes per fish per assay. The left panel shows the total number of freezing episodes per fish per assay. The right panel shows freezing events classified according to their outcome: "freezing episodes followed by normal return to swimming activity" or "freezing episodes immediately followed by hyperactive erratic bursts". **C,** Percentage of each freezing type per animal per assay. **D-G,** Seizure score over time during 20-min exposure to increasing concentrations of PTZ (0.5, 1, 5, and 10 mM) in wt and *nrxn2a^{tmr}2b^{tmr}*. Data are shown as mean ± SEM. Mean seizure score was analysed using aligned rank transform ANOVA with genotype and time as factors and fish ID as a repeated measure. Post-hoc comparisons between genotypes at each time point were performed using Holm correction for multiple testing. (*$p < 0.05$, **** $p < 0.0001$).

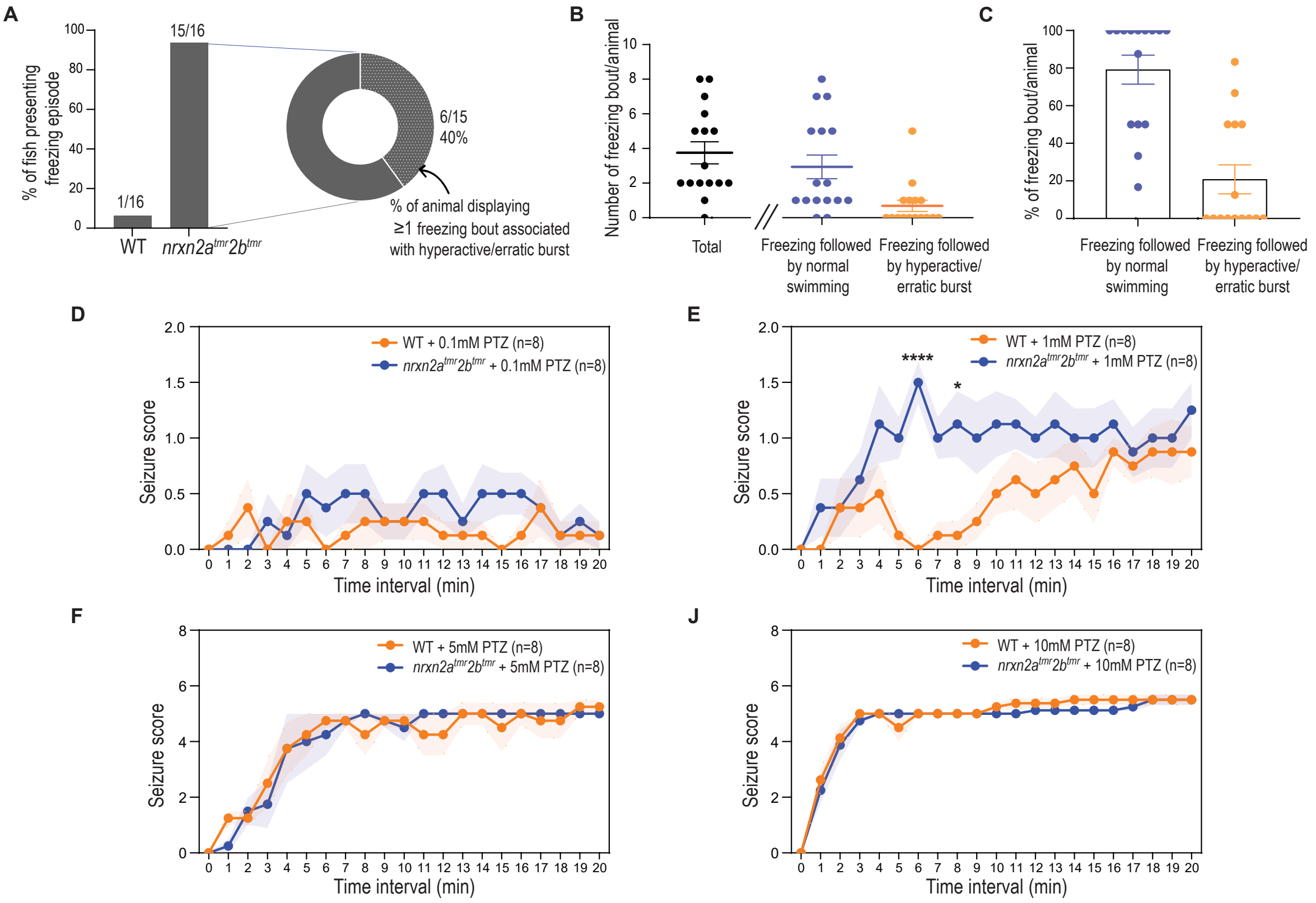

Supplemental figure S4. ***nrxn1*** **locus deletion results in reduced aggressiveness (dyadic fighting test).** Two size-match male fish of each genotype group were placed in a behavioral tank separated by an opaque divider and acclimated for 24h before the test to induce aggressiveness. After the 24-h acclimation, the divider was removed, and the fish were allowed to interact freely for 30 min. The number of bites was quantified using the manual scoring function of EthoVision XT software (Ver. 18.0.1803, Noldus) and plotted using GraphPad Prism (version 9.0.0). **A**, Number of bites recorded per minute during the 30-minute interaction. **B**, Average number of bites over the entire 30-minute recording period (n=4; 2 fish per point). Data are shown as mean ± SEM. Comparisons between two groups were performed using a two-tailed Mann-Whitney test ($p < 0.05$).

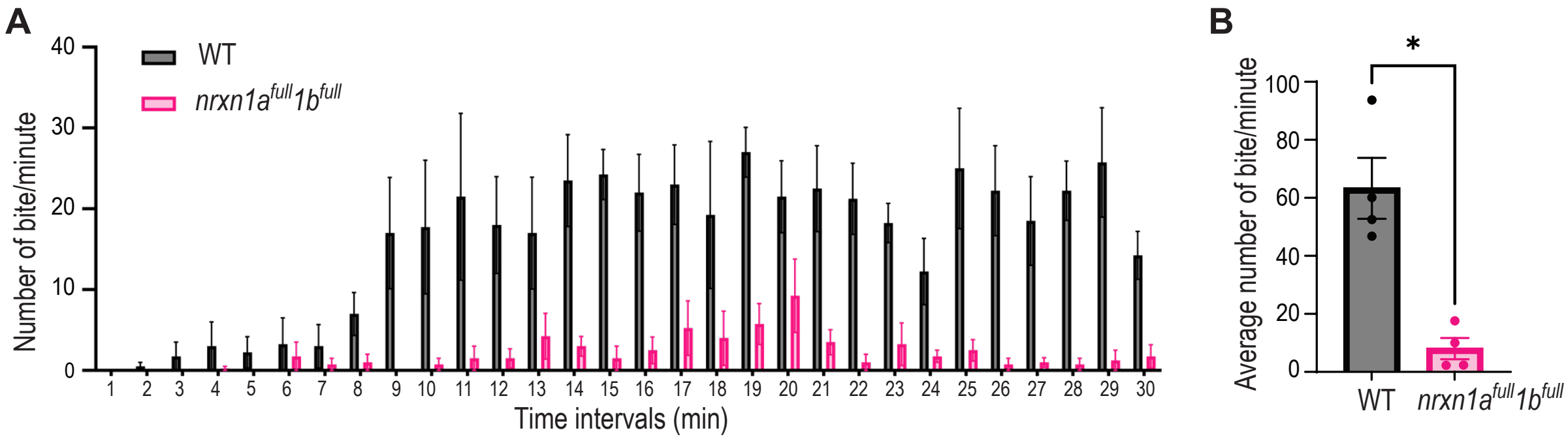

Supplemental Video S1. *nrxn2* mutants exhibit a near-constant bottom-dwelling behaviour under standard facility conditions. This 4-minute recording shows the different *neurexin* lines within their respective home tanks, filmed immediately after removal from the facility rack.
Available online to download

Supplemental Video S2. Representative (4× speed) video recordings of the Novel Tank diving Tests performed in this study.
Available online to download

Supplemental Video S3. Representative video recordings of 2 types of freezing events observed during Novel Tank diving Test
Available online to download

Supplemental Video S4. Representative video recordings of the affiliative social behavior assays conducted in this study.
Available online to download

Supplemental Video S5. Representative video recordings of the mirror biting assays conducted in this study.
Available online to download

Supplemental Files S1. Zip file containing SnapGene files of the different neurexin zebrafish genes with guides, primers and deletions generated in this study.
Available online to download

Supplemental Files S2. Raw data and Scripts associated with this study.
Available online to download

Supplemental Files S3. Statistical calculations and details associated with the figures presented in the study.
Available online to download
